# Substrate promiscuity of polyketide synthase enables production of tsetse fly attractants 3-ethylphenol and 3-propylphenol by engineering precursor supply in yeast

**DOI:** 10.1038/s41598-020-66997-5

**Published:** 2020-06-19

**Authors:** Julia Hitschler, Martin Grininger, Eckhard Boles

**Affiliations:** 1 Institute of Molecular Biosciences, Faculty of Biological Sciences, Goethe University Frankfurt, Max-von-Laue Straße 9, 60438 Frankfurt am Main, Germany; 2 Institute of Organic Chemistry and Chemical Biology, Buchmann Institute for Molecular Life Sciences, Goethe University Frankfurt, Max-von-Laue-Str. 15, 60438 Frankfurt am Main, Germany

**Keywords:** Applied microbiology, Molecular biology, Metabolic engineering, Applied microbiology, Metabolic engineering

## Abstract

Tsetse flies are the transmitting vector of trypanosomes causing human sleeping sickness and animal trypanosomiasis in sub-saharan Africa. 3-alkylphenols are used as attractants in tsetse fly traps to reduce the spread of the disease. Here we present an inexpensive production method for 3-ethylphenol (3-EP) and 3-propylphenol (3-PP) by microbial fermentation of sugars. Heterologous expression in the yeast *Saccharomyces cerevisiae* of phosphopantetheinyltransferase-activated 6-methylsalicylic acid (6-MSA) synthase (MSAS) and 6-MSA decarboxylase converted acetyl-CoA as a priming unit via 6-MSA into 3-methylphenol (3-MP). We exploited the substrate promiscuity of MSAS to utilize propionyl-CoA and butyryl-CoA as alternative priming units and the substrate promiscuity of 6-MSA decarboxylase to produce 3-EP and 3-PP in yeast fermentations. Increasing the formation of propionyl-CoA by expression of a bacterial propionyl-CoA synthetase, feeding of propionate and blocking propionyl-CoA degradation led to the production of up to 12.5 mg/L 3-EP. Introduction of a heterologous ‘reverse ß-oxidation’ pathway provided enough butyryl-CoA for the production of 3-PP, reaching titers of up to 2.6 mg/L. As the concentrations of 3-alkylphenols are close to the range of the concentrations deployed in tsetse fly traps, the yeast broths might become promising and inexpensive sources for attractants, producible on site by rural communities in Africa.

## Introduction

Kairomones are messenger substances for the transfer of information between different species, which are beneficial for the receiving organism only. 3-alkylphenols (3-methyl-, 3-ethyl- and 3-propylphenol) are kairomones, e.g. contained in cattle urine^1^, and attract tsetse flies that feed on the blood of vertebrate animals and humans. Tsetse flies, *Glossina sp*., inhabit sub-saharan Africa and are the main transmitting vector of trypanosomes, unicellular parasitic flagellate protozoa causing the widespread diseases human sleeping sickness and animal trypanosomiasis^2,3^. Animal trypanosomiasis considerably limits agricultural production and causes rural poverty by increasing livestock morbidity and mortality^4^. Human sleeping sickness is fatal if untreated and severely impacts human health especially in rural communities with inefficient health care provision^3^. An attractive way to combat the trypanosome transmission is to reduce the size of populations of tsetse flies. To do so, traps are impregnated with 3-alkylphenols among other compounds which serve as odour to attract the tsetse flies^1,5^. 3-Propylphenol (3-PP), optionally in combination with 3-methylphenol (3-MP), mainly attracts the tsetse fly species *G. pallidipes*, whereas 3-ethylphenol (3-EP) preferentially attracts *G. morsitans*^5,6^. Currently, 3-alkylphenols are mainly produced from fossil resources or are chemically synthesized e.g. from cashew nut shell liquids thereby relying on elaborate extraction procedures and expensive catalysts^7^.

In order to make 3-alkylphenols accessible for the poor rural communities in sub-saharan Africa, microbial fermentation offers an alternative method for the inexpensive and simple production of these compounds on site. Microbial fermentations are used since millennia e.g. for brewing beer and baking bread. Recent progress in metabolic engineering has fostered the development of microbial fermentation into a valid technology capable of producing a plethora of technologically relevant chemicals^8,9^. The yeast *Saccharomyces cerevisiae* is one of the most prominent microbes harnessed for fermentations. Its use as microbial production platform is advantageous, because it is well characterized, robust, simple to handle and easily genetically accessible^10–12^. Moreover, recombinant strains have been proven capable of fermenting agricultural waste materials, making compounds available at low costs^13^.

**Figure 1:**
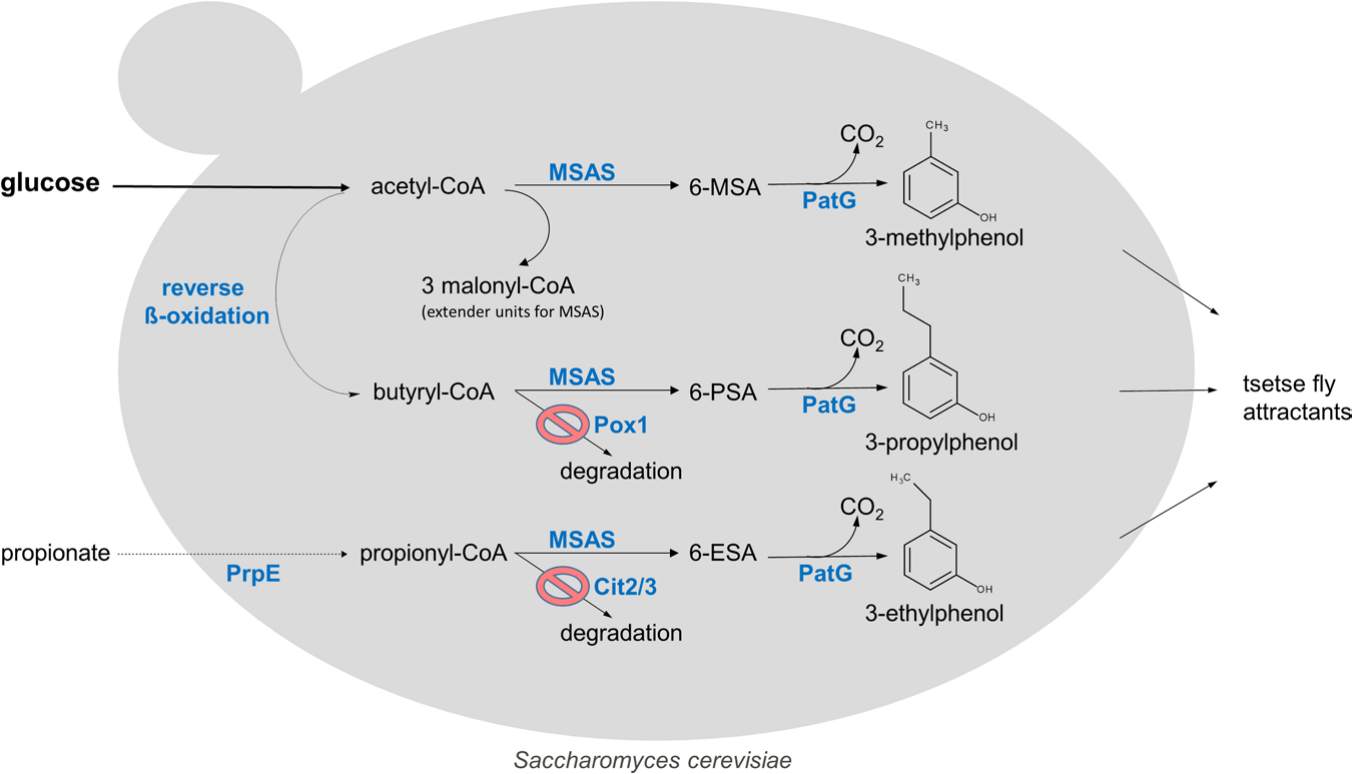
Metabolic pathways for 3-alkylphenol production in *S. cerevisiae*. In *S. cerevisiae* the heterologous polyketide synthase MSAS, activated by phosphopantetheinyl transferase (NpgA), catalyses the formation of 6-methylsalicylic acid (6-MSA) utilizing malonyl-CoA as extender unit and acetyl-CoA as priming unit. Intracellular propionyl-CoA can be increased by expression of a bacterial propionyl-CoA synthase (PrpE), propionate feeding and deletion of (methyl) citrate synthase genes *CIT2/3* to abolish its degradation. MSAS can then utilize propionyl-CoA as priming unit to catalyse the formation of 6-ethylsalicylic acid (6-ESA). The heterologous ‘reverse ß-oxidation’ pathway^21,22^ is providing the priming unit butyryl-CoA from acetyl-CoA for the formation of 6-propylsalicylic acid (6-PSA). Finally, 6-MSA decarboxylase (PatG) converts the 6-alkylsalicylic acids, 6-MSA, 6-ESA or 6-PSA, to their respective 3-alkylphenols (3-methylphenol, 3-ethylphenol or 3-propylphenol) that are valuable tsetse fly attractants.

We have recently developed an *S. cerevisiae* strain with a *de novo* 3-MP (*m*-cresol) production pathway from sugars^14^. In this recombinant yeast, a heterologous phosphopantetheinyltransferase (NpgA)-activated 6-methylsalicylic acid synthase (MSAS) utilizes acetyl-CoA as priming and malonyl-CoA as extender units to synthesize 6-methylsalicylic acid (6-MSA) that is further converted by 6-MSA decarboxylase (PatG) to 3-MP (Fig. 1). Since 3-EP and 3-PP show higher potential as tsetse fly attractants than 3-MP^5^, we focused on broadening this pathways for producing the 3-alkylphenols 3-EP and 3-PP.

The polyketide synthase MSAS natively functions with acetyl-CoA as priming unit for 6-MSA synthesis. It has been reported that enlarging the product spectrum is possible when priming MSAS with different priming units. For example, MSAS accepts propionyl-CoA and butyryl-CoA as priming units *in vitro* forming 6-ethylsalicylic acid (6-ESA) and 6-propylsalicylic acid (6-PSA), respectively^15,16^. Moreover, PatG was shown to decarboxylate 6-ESA to 3-EP *in vitro*^17^, while no data are available for the decarboxylation of 6-PSA.

From *in vitro* data, showing slow formation of 6-ESA from propionyl-CoA (13% of 6-MSA formation) and even slower formation of 6-PSA from butyryl-CoA (9% of 6-MSA formation)^15,16^, we expected that the competing formation of 6-MSA from acetyl-CoA dominates *in vivo*. The same limitations were anticipated to occur for the conversion of 6-ESA and 6-PSA by PatG again competing with 6-MSA. 3-MP production with yeast was not accompanied by noticeable 3-EP or 3-PP formation^14^, indicating that propionyl-CoA and butyryl-CoA are not available in high enough amounts in the yeast cells. We hypothesized that providing increased levels of cytosolic propionyl-CoA and butyryl-CoA could enable 3-EP and 3-PP production via MSAS and PatG in *S. cerevisiae*.

Propionyl-CoA is an intermediate in threonine catabolism in mitochondria but is probably directly degraded in the 2-methylcitrate cycle by 2-methylcitrate synthases Cit2 and Cit3^18,19^. Moreover, transport out of the mitochondria might further limit its accessibility for MSAS. To enhance propionyl-CoA levels in the yeast cells, we blocked its degradation and increased its production, finally leading to the formation of 3-EP (Fig. 1).

Butyryl-CoA in yeast cells might be derived from ß-oxidation of fatty acids in peroxisomes. However, normally it is further degraded to acetyl-CoA by fatty acyl-CoA oxidase Pox1 and ß-oxidation^20^. We increased butyryl-CoA levels by expressing a heterologous ‘reverse ß-oxidation’ pathway originally developed for *n*-butanol production from glucose^21,22^, leading to the production of 3-PP (Fig. 1).

Our data indicate that the promiscuities of MSAS and MSA decarboxylase can be harnessed for the *in vivo* production of various 3-alkylphenols, provided that the corresponding substrates are supplied in sufficient quantities.

## Material and Methods

### Strains and plasmids

Yeast strains and plasmids used in this study are described in Hitschler and Boles^14^ or are listed in Table 1. *S. cerevisiae* was cultivated in YPD medium (20 g/L peptone, 10 g/L yeast extract, 20 g/L glucose) from freshly streaked YPD agar plate cultures. For fermentations the medium was supplemented with 100 mM potassium phosphate (KP_i_) buffer (pH 6.5). Appropriate antibiotics (200 mg/L hygromycin or 200 mg/L G418) were added to media for plasmid maintenance. *Escherichia coli* DH10ß (Gibco BRL, Gaithersburg, MD) was grown in lysogeny broth (LB)-medium (10 g/L trypton, 5 g/L yeast extract, 5 g/L sodium chloride, pH 7.5) supplemented with appropriate antibiotics (100 mg/L carbenicillin, 50 mg/L kanamycin or 25 mg/L chloramphenicol) for plasmid maintenance and cloning.

**Table 1:** Plasmids and yeast strains used in this study. Genes from *Aspergillus nidulans* (An), *Aspergillus clavatus* (Ac), *Clostridium acetobutylicum* (Ca), *Penicillium patulum* (Pp), *Saccharomyces cerevisiae* (Sc), *Salmonella enterica serovar typhimurium* (St), *Treponema denticola* (Td) and codon-optimized genes (opt) are indicated by prefixes in superscript.

Plasmid	Plasmid based on	Relevant features	Reference
pUG6-H	—	*pBR322, hphNT1, Amp*^*r*^	^23^
pRS42K	—	*2 µ, kanMX, Amp*^*r*^	^24^
pRS72N	—	*2 µ, natMX, Amp*^*r*^	^24^
pRCC-K	—	*2 µ, kanMX, Amp*^*r*^*, pROX3-*^*opt*^*Cas9-tCYC1, pSNR52-gRNA*	^25^
pRS42K_prpE^783Δ^	pJHV1	*2 µ, kanMX, Amp*^*r*^*, pMET25, tCYC1, pTDH3-*^*Stopt*^*prpE*^*G783*^*-tPGK1*	This work
pJHV19	pRCC-K	*2 µ, kanMX, Amp*^*r*^*, pROX3-*^*opt*^*Cas9-tCYC1, pSNR52-gRNA* for *SFA1*	This work
pJHV54	pRCC-K	*2 µ, kanMX, Amp*^*r*^*, pROX3-*^*opt*^*Cas9-tCYC1, pSNR52-gRNA* for *CIT3*	This work
pJHV62	—	*ColE1, Amp*^*R*^*,ConL3*′*-pTDH3-*^*Tdopt*^*ter-tADH1 –ConRE‘*	This work
pJHV65	—	*ConLS‘-pPGK1p-*^*Sc*^*ERG10-tVMA16 -ConR1*′*-ConL1*′*-pCCW12-*^*Caopt*^*hbd-tIDP –ConR2*′*-ConL2*′*-pENO2-*^*Caopt*^*crt-tPGK1 –ConR3*′*-ConL3*′*-pTDH3-*^*Tdopt*^*ter-tADH1 –ConRE‘-natMX-LEU2 3*′*Hom-KanR-ColE1-LEU 5*′*Hom*	This work
pRS72N_ADY2	—	*2 µ, natMX, Amp*^*r*^*, pHXT7*^*-1–392*^*-*^*Sc*^*ADY2-tCYC1*	^26^
pRS72N_JEN1	—	*2 µ, natMX, Amp*^*r*^*, pHXT7*^*-1–392*^*-*^*Sc*^*JEN1-tCYC1*	^26^
SiHV110	—	*ConLS’-gfp-dropout-ConRE’-natMX-LEU2 3’Hom-KanR-ColE1-LEU 5’Hom*	This work (provided by Simon Harth)
pYTK3.41	—	*ColE1, Cam*^*R*^, ^*Tdopt*^*ter*	This work (provided by Fernando Garcés Daza)
pYTK3.43	—	*ColE1, Cam*^*R*^, ^*Caopt*^*crt*	This work (provided by Fernando Garcés Daza)
pYTK3.47	—	*ColE1, Cam*^*R*^, ^*Sc*^*ERG10*	This work (provided by Fernando Garcés Daza)
pYTK3.49	—	*ColE1, Cam*^*R*^, ^*Caopt*^*hbd*	This work (provided by Fernando Garcés Daza)
pYTK_Erg10	—	*ColE1, Amp*^*R*^*,ConLS‘-pPGK1-*^*Sc*^*ERG10-tVMA16 -ConR1‘*	This work (provided by Fernando Garcés Daza)
pYTK_Hbd	—	*ColE1, Amp*^*R*^*,ConL1*′*-pCCW12-*^*Caopt*^*hbd-tIDP –ConR2*′	This work (provided by Fernando Garcés Daza)
pYTK_Crt	—	*ColE1, Amp*^*R*^*,ConL2*′*-pENO2-*^*Caopt*^*crt-tPGK1 –ConR3*′	This work (provided by Fernando Garcés Daza)
pYTK01	—	*ColE1, Cam*^*R*^*, gfp-dropout*	^27^
pYTK05	—	*ColE1, Cam*^*R*^*, ConL3*	^27^
pYTK09	—	*ColE1, Cam*^*R*^*, TDH3p*	^27^
pYTK53	—	*ColE1, Cam*^*R*^*, ADH1t*	^27^
pYTK72	—	*ColE1, Cam*^*R*^*, ConRE*	^27^
pYTK95	—	*ColE1, Cam*^*R*^*, Amp*^*R*^*-ColE1*	^27^
pVS06	—	*CEN6ARS4, kanMX, Amp*^*r*^*, pHXT7*^*-1–392*^*-*^*Sc*^*ERG10-tVMA16, pPGK1-*^*Caopt*^*hbd-tEFM1, pTPI1-*^*Caopt*^*crt-tYHI9, pPYK1-*^*Tdopt*^*ter-tIDP1, pADH1-*^*Caopt*^*adhE2-tRPL3, pTDH3-*^*Ecopt*^*eutE-tRPL41B*	^22^
pRS62H_ter	—	*2 µ, natMX, Amp*^*r*^*, pHXT7*^*-1–392*^-^*Tdopt*^*ter-tFBA1*	^21^
pAB02	—	*2 µ, natMX, Amp*^*r*^*, pROX3-*^*opt*^*Cas9-tCYC1, pSNR52-gRNA* for *POX1*	This work (provided by Alexander Bissl)
pAB09	—	*2 µ, natMX, Amp*^*r*^*, pROX3-*^*opt*^*Cas9-tCYC1, pSNR52-gRNA* for *CIT2*	This work (provided by Alexander Bissl)
***S. cerevisiae*** **strain**	**Parent strain**	**Relevant features**	**Reference**
CEN.PK2-1C	—	*MATa leu2-3,112 ura3-52 trp1-289 his3-Δ1 MAL2–8*^*c*^ *SUC2*	^28^
JHY65	CEN.PK2-1C	*psfa1-sfa1Δ::pTDH3-*^*Stopt*^*prpE-tSFA1*	This work
JHY162	CEN.PK2-1C	*ura3::pPGK1-*^*Ppopt*^*MSAS-tCYC1, pHXT7*^*-1–392*^*-* ^*Anopt*^*npgA-tFBA1, pFBA1-*^*Acopt*^*patG-tADH1*	^14^
JHY164	CEN.PK2-1C	*cit2Δ*	This work
JHY174	JHY164	*cit2Δ cit3Δ*	This work
JHY175	CEN.PK2-1C	*cit3Δ*	This work
JHY179	JHY65	*psfa1-sfa1Δ::pTDH3-*^*Stopt*^*prpE-tSFA1 cit3Δ*	This work
JHY180	JHY179	*psfa1-sfa1Δ::pTDH3-*^*Stopt*^*prpE-tSFA1 cit3Δ cit2Δ*	This work
JHY185	JHY180	*psfa1-sfa1Δ::pTDH3-*^*Stopt*^*prpE-tSFA1 cit3Δ cit2Δ ura3::pPGK1-*^*Ppopt*^*MSAS-tCYC1, pHXT7*^*-1–392*^*-* ^*Anopt*^*npgA-tFBA1, pFBA1-*^*Acopt*^*patG-tADH1*	This work
JHY194	JHY162	*ura3::pPGK1-*^*Ppopt*^*MSAS-tCYC1, pHXT7*^*-1–392*^*-* ^*Anopt*^*npgA-tFBA1, pFBA1-*^*Acopt*^*patG-tADH1 leu2::pPGK1-*^*Sc*^*ERG10-tVMA16, pCCW12-*^*Caopt*^*hbd-tIDP, pENO2-*^*Caopt*^*crt-tPGK1, pTDH3-*^*Tdopt*^*ter-tADH1, pTEF-natMX-tTEF*	This work
JHY196	CEN.PK2-1C	*pox1Δ*	This work
JHY197	JHY174	*cit2Δ cit3Δ ura3::pPGK1-*^*Ppopt*^*MSAS-tCYC1, pHXT7*^*-1–392*^*-* ^*Anopt*^*npgA-tFBA1, pFBA1-*^*Acopt*^*patG-tADH1*	This work
JHY211	JHY196	*pox1Δ ura3::pPGK1-*^*Ppopt*^*MSAS-tCYC1, pHXT7*^*-1–392*^*-* ^*Anopt*^*npgA-tFBA1, pFBA1-*^*Acopt*^*patG-tADH1*	This work
JHY212	JHY211	*pox1Δ ura3::pPGK1-*^*Ppopt*^*MSAS-tCYC1, pHXT7*^*-1–392*^*-* ^*Anopt*^*npgA-tFBA1, pFBA1-*^*Acopt*^*patG-tADH1 leu2::pPGK1-*^*Sc*^*ERG10-tVMA16, pCCW12-*^*Caopt*^*hbd-tIDP, pENO2-*^*Caopt*^*crt-tPGK1, pTDH3-*^*Tdopt*^*ter-tADH1, pTEF-natMX-tTEF*	This work
JHY218	JHY65	*sfa1p-sfa1Δ::TDH3p-*^*Stopt*^*prpE-SFA1t ura3::pPGK1-*^*Ppopt*^*MSAS-tCYC1, pHXT7*^*-1–392*^*-* ^*Anopt*^*npgA-tFBA1, pFBA1-*^*Acopt*^*patG-tADH1*	This work
JHY229	JHY185	*psfa1-sfa1Δ::pTDH3-*^*Stopt*^*prpE-tSFA1 cit3Δ cit2Δ ura3::pPGK1-*^*Ppopt*^*MSAS-tCYC1, pHXT7*^*-1–392*^*-* ^*Anopt*^*npgA-tFBA1, pFBA1-*^*Acopt*^*patG-tADH1 pacs2-acs2Δ::pTEF-hphNT1-tCYC1*	This work

Other abbreviations: *hphNT1*: hygromycin resistance; *Amp*^r^: ampicillin resistance; *Cam*^*R*^: chloramphenicol resistance; *Kan*^*R*^: kanamycin resistance; *kanMX*: geneticin resistance; *natMX*: clonat resistance. If not stated otherwise, promoters (p) were taken 1-500 bp upstream and terminators (t) 1-300 bp downstream of respective open reading frames.

### Plasmid and strain construction

The codon-optimized DNA sequences, ^*opt*^*patG* (GeneBank accession number MK791645), ^*Ppopt*^*MSAS (*MK791642), ^*opt*^*npgA* (MK791644) and ^*opt*^*prpE* (MT219994), were obtained with the JCat tool^29^ and ordered as GeneArt Strings DNA fragments from Thermo Fischer Scientific. Genomic DNA of CEN.PK2-1C or plasmids were used as templates for PCR amplification of yeast open reading frames, promoters and terminators with 35 bp homologous overlaps. Primers and genes used in this study are described in Hitschler and Boles^14^, Schadeweg and Boles^21,22^ or are listed in Supplementary Tables S1 and S2.

Plasmid assembly in yeast via homologous recombination or in *E. coli* via Gibson assembly^30^ and plasmid propagation were conducted as described previously^14^. Genomic integrations into the *ura3* locus of CEN.PK2-1C were performed with the CRISPR/Cas9 system^25^ as described in Hitschler and Boles^14^. For deletions, CRISPR/Cas9 plasmids carrying the guide RNA (gRNA) for the specific deletion were amplified via PCR and assembled via Gibson. The donor DNA consisting of 40 bp upstream and 40 bp downstream sequences of the open reading frame were ordered as primers and were annealed to double-stranded DNA as described in Reifenrath and Boles^31^. However, for deletion of *ACS2* in the JHY185 strain a pUG6-based deletion cassette^23^ was amplified, conferring resistance to hygromycin and carrying 40 bp overhangs to the *ACS2* locus for integration via homologous recombination.

For genomic integration of the ‘reverse ß-oxidation’ pathway genes (Schadeweg and Boles) the Golden Gate system^27^ was utilized for construction of an integration vector. Part plasmids were obtained from Lee *et al*.^27^ or PCR fragments with part type specific overhangs and pYTK01 as backbone were assembled with *Esp3*I as described previously^27^ incubating the reaction mixture for 10 min at 37 °C, using 15 cycles of digestion and ligation (37 °C 2 min, 16 °C 5 min) and heat inactivating the enzymes at 60 °C for 10 min and 80 °C for 10 min and transformed into *E. coli*. Next cassette plasmids were formed carrying *Bsm*BI overhangs flanking the cassette for subsequent assembly of the integration plasmid. To build the cassette plasmid pJHV62, part plasmids pYTK3.41, pYTK05, pYTK09, pYTK053, pYTK72 and pYTK95 were assembled with *Bsa*I-HF. For assembly of the integration vector pJHV65 with *Esp3*I, the reaction mixture containing the cassette plasmids pYTK_ERG10, pYTK_hbd, pYTK_crt, pJHV62 and SiHV110 were incubated for 10 min at 37 °C, using 25 cycles of digestion and ligation (37 °C 1.5 min, 16 °C 3 min), 37 °C for 5 min and heat inactivating the enzymes at 50 °C for 5 min and 80 °C for10 min and transformed into *E. coli*. The integration vector pJHV65 was digested with *Not*I and 500 bp homologous sequences to the upstream and downstream region of *LEU2* flanked the integration cassette and *natMX* cassette for homologous recombination and selection in yeast. After transformation of yeast with respective DNA fragments according to Gietz and Schiestl^32^, cells were grown on selective YPD agar plates.

### Cell cultivation

Cells were cultivated in 150 mL YPD medium supplemented with corresponding antibiotics and buffered with 100 m potassium phosphate buffer (KP_i_) at pH 6.5 to avoid unwanted effects of weak acids. Overnight cultures were harvested in exponential phase and utilized for inoculation of 25 mL KP_i_ buffered YPD medium (pH 6.5) to an optical density (OD_600 nm_) of 4 or more. For consumption or biotransformation experiments, 10 mM butyrate or propionate were added, respectively. Cultures were shaken at 180 rpm at 30 °C for 144 h in a waterbath (Memmert, Germany) or in a 30 °C container to prevent inhalation of 3-alkylphenols.

### Growth and metabolite analysis

The spectrophotometer Ultrospec 2100 pro (GE Healthcare, USA) was utilized to follow cell growth at an optical density of 600 nm. Culture supernatants for HPLC analysis of 3-alkylphenol formation were prepared as described previously^14^ and analysis was performed via HPLC (Dionex) with an Agilent Zorbax SB-C8 column (4.6 ×150 mm, 3.5 µm) at 40 °C and at a flow rate of 1 mL/min. 3-methylphenol and 3-ethylphenol were separated by the same gradient of solvent A (0.1% (*v/v*) formic acid in ddH_2_O) and solvent B (0.1% (*v/v*) formic acid in acetonitrile) mentioned before^14^. The same gradient applied for 3-propylphenol analysis with the exception that the gradient stayed at 40% B for 5 min before it switched to 100% B to prolong the separation before the washing step. The 3-alkylphenols were detected at 270 nm in an UV detector (Dionex UltiMate 3000 Variable Wavelength Detector). For quantification and calibration, 3-alkylphenol standards were prepared in ddH_2_O from *m*-cresol purchased from Carl Roth (9269.1), 3-ethylphenol from Sigma-Aldrich (210-627-3) and 3-propylphenol from Alfa Aesar (621-27-2).

For propionate analysis 50 µL 50% (*w/v*) sulfosalicylic acid was added to 450 µL culture supernatant. Samples were analysed in the HPLC equipped with the ion exchange column HyperREZ XP Carbohydrate H + (7.7 × 300 mm, 8 µm) and a refractive index detector (Thermo Shodex RI-101). The metabolites were separated with 5 mM sulfuric acid as liquid phase at a flow rate of 0.6 mL/min and 65 °C. For quantification, propionate standards of different concentrations were prepared in ddH_2_O from propionic acid purchased from Carl Roth (6026.2). Data analysis and graphing were performed utilizing the software Prism 5 (Graphpad).

## Results and Discussion

### Production of 3-ethylphenol from propionyl-CoA

#### Propionate supplementation enables 3-ethylphenol formation

We aimed to synthesize 3-ethylphenol (3-EP) *in vivo* from glucose via MSAS and MSA decarboxylase by provision of propionyl-CoA as a priming unit for MSAS. This approach relies on high intracellular levels of propionyl-CoA to successfully compete with acetyl-CoA as the cognate priming unit of MSAS. In principle, enhanced propionyl-CoA concentrations can be achieved by either manipulating endogenous pathways leading to propionyl-CoA, feeding of propionate to the cells, blocking of propionyl-CoA degradation and/or enhancing direct propionyl-CoA synthesis. In yeast cells, endogenous pathways for generation of propionyl-CoA exist. For example, propionyl-CoA is an intermediate in yeast threonine catabolism which takes place in the mitochondria^18,19^. Threonine degradation to propionyl-CoA is initiated by threonine deaminase, catalyzing the conversion of threonine to 2-ketobutyrate. The 2-ketoacid dehydrogenase complex can then catalyze the oxidative decarboxylation of 2-ketobutyrate to propionyl-CoA. In addition, the acetyl-CoA synthases of *S. cerevisiae* are able to convert externally supplied propionate to propionyl-CoA^33^.

**Figure 2:**
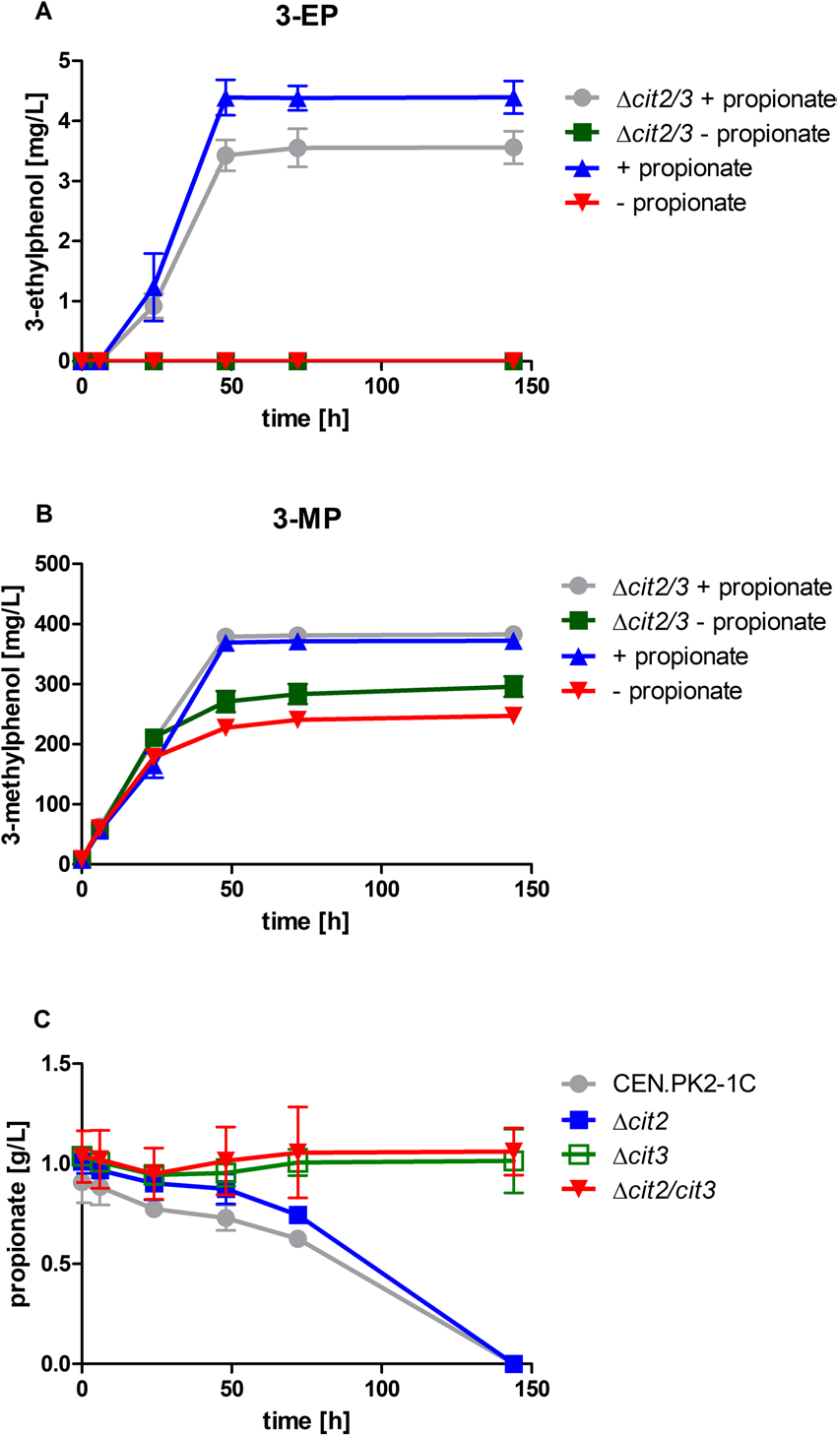
Effect of deletion of methylcitrate synthase genes *CIT2* and *CIT3* on 3-ethylphenol (**A**) and 3-methylphenol (**B**) formation with or without supplementation of external propionate and on propionate consumption (**C**). CEN.PK2-1C expressing the 3-methylphenol pathway (JHY162) (^*Ppopt*^*MSAS*, ^*opt*^*npgA* and ^*opt*^*patG*^14^) and with the *Δcit2Δcit3* double deletion (JHY197) were utilized for high-OD fermentations (starting OD = 5.0) at 30 °C in KP_i_ buffered YPD medium (pH 6.5) with or without supplementation of 10 mM propionate. Propionate consumption was followed in *S. cerevisiae* wild-type strain CEN.PK2-1C and deletion strains that either had peroxisomal (*Δcit2*), mitochondrial (*Δcit3*) or both methylcitrate synthases (*Δcit2/Δcit3*) deleted and were cultured (starting OD = 4) at 30 °C in KP_i_ buffered YPD medium (pH 6.5) supplemented with 10 mM propionate. Culture supernatants were analysed via HPLC for 3-alkylphenol production and propionate. Error bars represent standard deviations of biological duplicates.

We first wanted to test whether it is possible to rely on endogenous pathways to provide enough propionyl-CoA for 3-EP production. For this, we utilized the 3-methylphenol (3-MP) production strain JHY162 from our previous work^14^. Strain JHY162 expresses ^*Ppopt*^*MSAS*, ^*opt*^*npgA* and ^*opt*^*patG* under control of the strong constitutive p*PGK1*, p*HXT7*^*-1–392*^ and p*FBA1* promoters, respectively, which were stably integrated in the *ura3* locus of the *S. cerevisiae* strain CEN.PK2-1C^14^. A high-OD fermentation (starting OD = 5) in KP_i_ buffered YPD at pH 6.5 revealed that strain JHY162 only produced 3-MP (296 mg/L) but 3-EP could not be detected in the supernatants of the cultures (Fig. 2). This indicated that propionyl-CoA, either generated by threonine catabolism^18,19^ or other endogenous pathways, is not available at sufficient concentrations to outcompete acetyl-CoA for conversion by MSAS. Moreover, endogenous propionyl-CoA might be directly degraded in the 2-methylcitrate cycle by 2-methylcitrate synthases Cit2 and Cit3^18,19,34^.

Nevertheless, when adding 10 mM propionate to the medium, the same strain (JHY162) produced up to 4.4 mg/L 3-EP (Fig. 2A), indicating synthesis of propionyl-CoA and its conversion by MSAS and PatG to 3-EP. We assume that propionate is converted to propionyl-CoA by the endogenous acetyl-CoA synthetases of *S. cerevisiae*^33^. In spite of this success, the main product was still 3-MP which accumulated up to 372 mg/L (Fig. 2B) reflecting the preference of MSAS for acetyl-CoA as priming unit and of PatG for MSA^15–17^. Surprisingly, also 3-MP formation was stimulated by the addition of propionate.

To increase propionyl-CoA levels as the priming unit for 3-EP formation, we aimed at blocking propionyl-CoA degradation which is mediated by 2-methylcitrate synthases. It was shown that abolishment of 2-methylcitrate synthase activity in a Δ*cit2* Δ*cit3* deletion strain prevented propionate degradation^34^. To confirm this in our strains, we deleted *CIT2* or *CIT3* individually or both together in CEN.PK2-1C and performed fermentations with a starting OD of 4 in KP_i_ buffered YPD medium at pH 6.5 supplemented with about 10 mM propionate. Indeed, the Δ*cit2* Δ*cit3* strain JHY174 did not consume any propionate over 144 hours. The single knock-out strains revealed that even a *cit3* deletion alone (JHY175) is enough to abolish propionate degradation. Externally added propionate was completely consumed by the CEN.PK2-1C wildtype strain and the Δ*cit2* strain JHY164 (Fig. 2C).

Although deletion of only *CIT3* already abolished propionate degradation, we used the Δ*cit2* Δ*cit3* double deletion strain to test an influence on 3-EP formation. We expressed ^*Ppopt*^*MSAS*, ^*opt*^*npgA* and ^*opt*^*patG* together under control of the strong constitutive p*PGK1*, p*HXT7*^*-1–392*^ and p*FBA1* promoters, respectively, integrated into the *ura3* locus of strain JHY174 (resulting in strain JHY197), and performed high-OD fermentations (starting OD = 5.0) in KP_i_ buffered YPD at pH 6.5. Strain JHY197 showed a slightly better growth (not shown) and 3-MP production (296 mg/L) compared to JHY162 (247 mg/L) (Fig. 2B). However, obviously deletion of *CIT2* and *CIT3* was not sufficient to provide enhanced endogenous propionyl-CoA levels for 3-EP formation (Fig. 2A). Even when supplemented with 10 mM propionate in the medium, blocked propionyl-CoA degradation did not further increase 3-EP formation (up to 3.6 mg/L in JHY197) (Fig. 2A). Interestingly, also in the *cit2/3* deletion strain 3-MP formation was somehow stimulated by the addition of propionate. The results indicate that degradation of propionyl-CoA is not limiting 3-EP formation in the yeast cells.

#### A heterologous propionyl-CoA synthetase increased 3-EP formation

**Figure 3:**
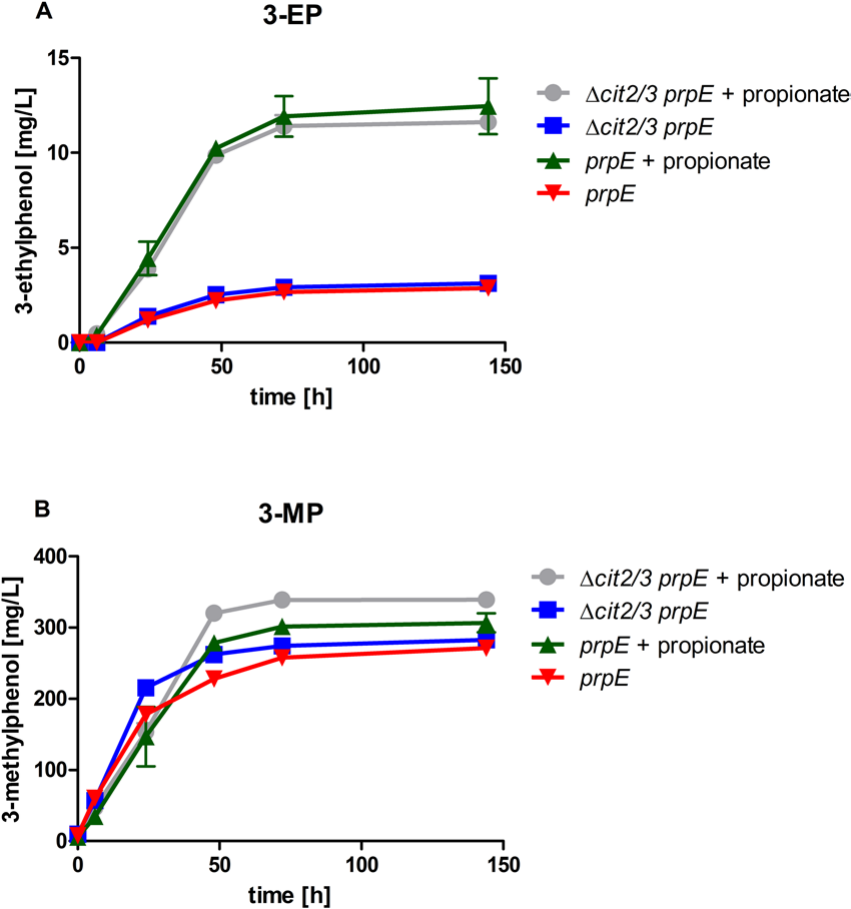
Influence of overexpression of a propionyl-CoA synthetase on 3-ethylphenol (**A**) and 3-methylphenol (**B**) formation with and without supplementation of external propionate. Yeast strains CEN.PK2-1C expressing the 3-methylphenol pathway (^*Ppopt*^*MSAS*, ^*opt*^*npgA* and ^*opt*^*patG*^14^*)* and additionally the propionyl-CoA synthase ^*opt*^*prpE*, with or without the *Δcit2Δcit3* double deletion (strains JHY185 and JHY218, respectively), were inoculated at an OD of 5 and cultivated for 144 h in KP_i_ buffered YPD medium (pH 6.5) with or without supplementation of 10 mM propionate. Culture supernatants were analysed via HPLC for 3-alkylphenol production. Error bars represent standard deviations of biological duplicates.

In order to provide additional propionyl-CoA, we expressed the codon-optimized propionyl-CoA synthetase gene ^*opt*^*prpE* from *Salmonella typhimurium*^35^ under control of the strong promoter p*TDH3*, integrated into the *sfa1* locus in both strains JHY162 and JHY197 (resulting in strains JHY218 and JHY185, respectively). Fermentations were performed as described above. 3-MP production with JHY218 and JHY185 was not significantly influenced compared to the strains without ^opt^PrpE (Fig. 3B). When the medium was supplemented with 10 mM propionate, 3-EP production noticeably increased to titers of up to 12.5 mg/L with strain JHY218 and 11.6 mg/L with strain JHY185 (Fig. 3A). This result demonstrates that normally endogenous yeast propionyl-CoA synthetase activity is limiting 3-EP formation. Moreover, as both strains – with or without the 2-methylcitrate cycle – produced comparable amounts of 3-EP it confirms that degradation of propionyl-CoA is not limiting 3-EP production.

Interestingly, expression of ^*opt*^*prpE* led to the formation of 3-EP even without externally added propionate with both strains (Fig. 3A) (3.1 mg/L with JHY185 and 2.9 mg/L with JHY218 after 144 hours). This indicates an endogenous unknown source of propionate. We speculate that this propionate derives from endogenous propionyl-CoA which is hydrolyzed (unspecifically) by thioesterases. Obviously, hydrolyzation of propionyl-CoA to propionate is very efficient and the released propionate needs to be reactivated to propionyl-CoA by a highly active propionyl-CoA synthetase. In this regard it is revealing that also feeding threonine (20 g/L), which is degraded to propionyl-CoA, increased 3-EP production only in the presence of the propionyl-CoA synthetase PrpE (up to 14.3 mg/L 3-EP) (Supplementary Fig. S1). In the absence of PrpE threonine feeding resulted only in 0.18 mg/L 3-EP. This result further confirms the intermediate formation of propionate.

#### Deletion of endogenous acetyl-CoA synthetase to change precursor ratio

All the strains producing 3-EP still produced much higher amounts of 3-MP (Figs. 2B and 3B). The 3-EP/3-MP ratio is influenced by (i) the substrate preferences of acyl-CoA synthetases, MSAS and MSA decarboxylase and (ii) the ratio of intracellular propionate/acetate, propionyl-CoA/acetyl-CoA respectively ESA/MSA. Acetyl-CoA is the preferred priming unit of MSAS and is an essential metabolite in yeast produced from acetate which is an intermediate of yeast carbon metabolism. Moreover, acetyl-CoA is even necessary for 3-EP formation as it is the substrate of acetyl-CoA carboxylase for the synthesis of the extender unit malonyl-CoA, the second substrate of MSAS.

Acetyl-CoA in yeast is mainly produced by acetyl-CoA synthetases Acs1 and Acs2^33^. As the additional expression of the bacterial propionyl-CoA synthase led to a noticeably increase in 3-EP formation, we aimed at increasing the ratio of 3-EP/3-MP by replacing the acetyl-CoA synthetase of yeast with the propionyl-CoA synthetase of *S. typhimurium*. We thought this is possible because the propionyl-CoA synthetase is known to be able to synthesize also acetyl-CoA^36^. Accordingly, feeding of propionate should increase the ratio of propionyl-CoA/acetyl-CoA.

Acetyl-CoA synthetase in yeast is encoded by the glucose-repressed *ACS1* gene and by *ACS2*^33^. On medium with glucose as the carbon source, the *ACS2* gene is essential for the production of acetyl-CoA and for growth. We deleted *ACS2* in strain JHY185 expressing the propionyl-CoA synthetase gene of *S. typhimurium* (resulting in strain JHY229). As JHY229 could grow on glucose this confirmed that the propionyl-CoA synthetase is able to provide acetyl-CoA. Interestingly, in fermentations in the absence of external propionate JHY229 produced slightly more 3-EP than the parent strain JHY185 (5.4 mg/L compared to 3.5 mg/L), indicating that the propionyl-CoA/acetyl-CoA ratio indeed might be increased. However, when the medium was supplemented with 10 mM propionate strain JHY229 did no longer grow nor produced any 3-MP or 3-EP (Supplementary Fig. S2). These results suggest that the intracellular propionate/acetate ratio became too high, and as the propionyl-CoA synthetase prefers propionate as its substrate the essential conversion of acetate to acetyl-CoA was blocked by propionate. To conclude, although the increase of the propionyl-CoA/acetyl-CoA ratio is a possible approach to improve production of 3-EP finding a suitable balance of propionyl-CoA to acetyl-CoA concentrations seems to be a difficult task.

### Production of 3-propylphenol from butyryl-CoA

#### Butyrate feeding is not sufficient for 3-propylphenol formation

3-Propylphenol (3-PP) is another promising attractant for tsetse flies^5^. In principle, it can be formed from butyryl-CoA as a priming unit of MSAS followed by decarboxylation of the formed 6-propylsalicylic acid (PSA). It has already been shown *in vitro* that MSAS can use butyryl-CoA as a substrate although conversion to PSA proceeds with 9% of the activity with acetyl-CoA^15,16^. The MSA decarboxylase PatG has not yet been tested for its activity with PSA.

In order to enable 3-PP production in *S. cerevisiae*, the priming unit butyryl-CoA is required in sufficient amounts. As shown above the endogenous acyl-CoA synthetases are able to convert propionate to propionyl-CoA. Moreover, Luo *et al*.^37^ recently even demonstrated the conversion of exogenously supplied hexanoic acid to hexanoyl-CoA for the production of olivetolic acid. Therefore, we tested whether the supplementation of 10 mM butyrate in KPi buffered YPD medium can provide enough butyryl-CoA for 3-PP formation via MSAS and MSA decarboxylase. However, high-OD fermentations of strain JHY162 expressing ^*Ppopt*^*MSAS*, ^*opt*^*npgA* and ^*opt*^*patG* under control of the strong constitutive p*PGK1*, p*HXT7*^*-1–392*^ and p*FBA1* promoters, respectively, did not result in any 3-PP formation (Supplementary Fig. S3A). Butyrate supplementation did not influence 3-MP production (Supplementary Fig. S3B).

To exclude the possibility that the formed butyryl-CoA is rapidly degraded via ß-oxidation, we deleted the fatty acyl-CoA oxidase encoding gene *POX1* in CEN.PK2-1C to abolish ß-oxidation^20^, and integrated the heterologous 3-MP production pathway as above, resulting in strain JHY211. For improved uptake of butyrate we transformed JHY211 with multi-copy plasmids overexpressing the endogenous monocarboxylic acid transporters Jen1 or Ady2^38–40^ under control of the strong p*HXT7*^*-1–392*^ promoter or an empty vector as control. However, high-OD fermentations in KPi buffered YPD medium supplemented with 10 mM butyrate did not result in any 3-PP formation in any of the strains (Supplementary Fig. S3C). These results indicate that either butyryl-CoA concentrations cannot be enhanced to levels at which it can compete with acetyl-CoA for priming MSAS or that MSAS and/or MSA decarboxylase are not able to convert butyryl-CoA and/or PSA, respectively, *in vivo*.

#### Heterologous ‘reverse ß-oxidation’ pathway supplies enough butyryl-CoA as precursor for 3-propylphenol production

Recently, our group established a ‘reverse ß-oxidation’ pathway for efficient *n*-butanol production from acetyl-CoA in yeast^21,22^. This pathway forms butyryl-CoA as an intermediate. In the pathway, two acetyl-CoA are condensed by endogenous thiolase Erg10 to acetoacetyl-CoA, which is then converted to 3-hydroxybutyryl-CoA and crotonyl-CoA by hydroxybutyryl-CoA dehydrogenase Hbd and crotonase Crt from *Clostridium acetobutylicum* and finally reduced to butyryl-CoA by trans-2-enoyl-CoA reductase Ter from *Treponema denticola*. To use the pathway for 3-PP production, we integrated the genes required up to butyryl-CoA formation (*ERG10* from *S. cerevisiae*, codon-optimized ^*opt*^*hbd* and ^*opt*^*crt* from *C. acetobutylicum* and ^*opt*^*ter* from *T. denticol*a) under control of the strong constitutive promoters p*PGK1*, p*CCW12*, p*ENO2*, p*TDH3*, respectively, into the *leu2* locus of yeast strains JHY162 and JHY211, resulting in strains JHY194 and JHY212, respectively.

**Figure 4:**
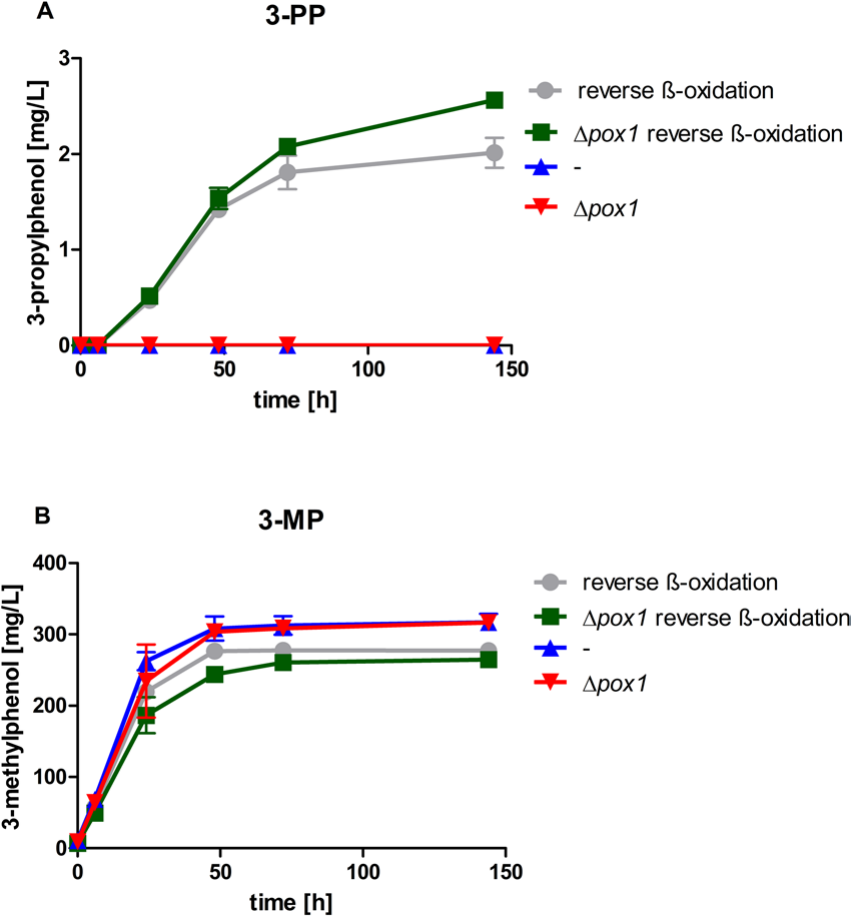
3-Propylphenol formation via ‘reverse ß-oxidation’. 3-Propylphenol (**A**) and 3-methylphenol production (**B**) was measured in culture supernatants of CEN.PK2-1C expressing the 3-methylphenol pathway (^*Ppopt*^*MSAS*, ^*opt*^*npgA* and ^*opt*^*patG*^14^) with or without additional expression of the ‘reverse ß-oxidation’ pathway (*ERG10*, ^*opt*^*hbd*, ^*opt*^*crt* and ^*opt*^*ter*^21,22^) (strains 194 and 162, respectively), and with additional *pox1* deletion (strains JHY212 and JHY211, respectively). High-OD fermentations (starting OD = 5) were performed in biological duplicates at 30 °C in KP_i_ buffered YPD medium at pH 6.5. Culture supernatants were analysed via HPLC for 3-alkylphenol production. Error bars represent standard deviations.

High-OD fermentations of JHY194 and JHY212 and their parent strains as controls were performed in KP_i_ buffered YPD (pH 6.5) at 30 °C. As observed before, strain JHY162, with the 3-methylphenol production pathway, and JHY211, with additional *pox1* deletion, were unable to produce 3-PP (Fig. 4A). However, expression of the ‘reverse ß-oxidation’ pathway resulted in up to 2 mg/L 3-PP formation with strain JHY194 (Fig. 4A). The highest 3-PP titer (2.6 mg/L) was achieved when *POX1* was additionally deleted (strain JHY212), indicating that butyryl-CoA degradation was partially limiting 3-PP production. As expected, 3-methylphenol was still produced in high amounts by all strains (Fig. 4B).

The ‘reverse ß-oxidation’-based *n*-butanol production was limited by the trans-2-enoyl-reductase Ter, and additional ^*Tdopt*^*ter* overexpression improved the final butanol titers^21^. To test whether Ter might also limit 3-PP formation, ^*Tdopt*^*ter* was additionally overexpressed from a multi-copy plasmid under control of the strong constitutive p*HXT7*^*-1–392*^ promoter in JHY194 and JHY212 and high-OD fermentations (starting OD = 5) were performed. However, additional ^*Tdopt*^*ter* overexpression did not increase 3-PP titers (1.1 mg/L and 0.7 mg/L with or without Ter overexpression in JHY194 and JHY212, respectively) indicating that other factors limit 3-PP production.

To conclude, obviously the ‘reverse ß-oxidation’ pathway provides more butyryl-CoA than exogenous addition of butyrate, and these levels are high enough to compete at least partially with acetyl-CoA and to be transformed into 3-PP.

## Conclusions

In this work we show that yeast engineered to provide increased intracellular formation of propionyl-CoA or butyryl-CoA and expressing MSAS and MSA decarboxylase can be exploited to produce 3-EP and 3-PP from sugars. The approach is based on the broad substrate tolerance of MSAS and MSA decarboxylase, shown before *in vitro*. In spite of this success, 3-MP derived from acetyl-CoA as the preferred priming substrate of MSAS and decarboxylation of the intermediate 6-MSA by MSA decarboxylase remained the main product of the engineered strains. Acetyl-CoA cannot be eliminated as it is an essential metabolite and is required for the production of malonyl-CoA as the elongation substrate of MSAS. Therefore, further approaches to increase production of 3-EP and 3-PP will require the engineering of the substrate specificity of MSAS. Since all the enzymatic domains of MSAS can essentially account for reduced rates in the turnover of substrates with elongated alkyl moiety, an elaborate engineering strategy is necessary including mutations of binding sites and swaps of catalytic domains^41^. A corresponding engineering of the MSA decarboxylase will be necessary.

Concerning the use of the 3-alkylphenols (3-MP, 3-EP, 3-PP) as baits in tsetse fly traps the titers achieved in our work are close to the natural concentrations in cattle urine (50 mg/L 3-MP, 5.5 mg/L 3-EP and 12.5 mg/L 3-PP) and the concentrations deployed in tsetse fly traps^1,5^. However, as higher concentrations improved catch rates^5^ and 3-EP and 3-PP are more effective than 3-MP, MSAS and MSA decarboxylase engineering might be useful to improve the effects. It remains to be tested whether it will be possible to simply use the whole yeast cultures, yeast extracts or supernatants to prepare the traps. Our work is a first step in facilitating the preparation of the traps by the simple and direct “brewing” of 3-alkylphenols. As they shall be produced locally by poor rural communities it is desirable to use waste residues from agriculture, food or feed as suitable substrates. This might require further engineering of the yeasts for utilisation of substrates deriving from materials such as lignocellulosic biomass, pectin or fats.

Apart from being used as tsetse fly attractants alkylphenols are also valuable organic industrial chemicals used e.g. in the production of lubricating oil additives and as surface-active substances in cleaning products. However, to replace or supplement these mainly fossil resources-derived alkylphenols the fermentative production process still needs to be improved considerably.

## Supplementary information

Supplementary information.

## Data Availability

Materials and data are made available on request.
