# Supplementary material for: Substrate promiscuity of polyketide synthase enables production of tsetse fly attractants 3-ethylphenol and 3-propylphenol by engineering precursor supply in yeast
Source: Sci Rep. 2020 Jun 19;10:9962. doi: 10.1038/s41598-020-66997-5 (PMC7305150; doi:10.1038/s41598-020-66997-5)
Supplement: Supplementary file 1 — Supplementary information. [file 41598_2020_66997_MOESM1_ESM.pdf]

Supplementary Material

Substrate promiscuity of polyketide synthase enables production of tsetse fly attractants 3-ethylphenol and 3-propylphenol by engineering precursor supply in yeast

Julia Hitschler[1], Martin Grininger[2] and Eckhard Boles[1]*

[1]Institute of Molecular Biosciences, Faculty of Biological Sciences, Goethe University Frankfurt, Max-von-Laue Straße 9, 60438 Frankfurt am Main, Germany

[2]Institute of Organic Chemistry and Chemical Biology, Buchmann Institute for Molecular Life Sciences, Cluster of Excellence „Macromolecular Complexes“, Goethe University Frankfurt, Max-von-Laue-Str. 15, 60438 Frankfurt am Main, Germany

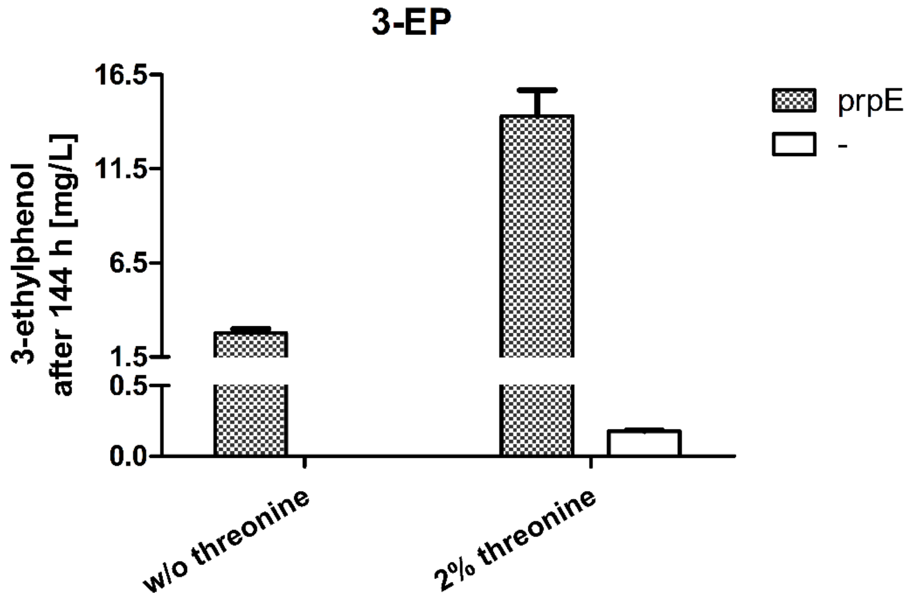

Figure S1. Influence of overexpression of a propionyl-CoA synthetase on 3-ethylphenol formation with and without supplementation of external threonine. Yeast strain CEN.PK2-1C with the *Δcit2Δcit3* double deletion expressing the 3-methylphenol pathway (^{Ppopt}*MSAS*, ^{opt}*npgA* and ^{opt}*patG*[14]) and with or without the propionyl-CoA synthase ^{opt}*prpE* (strains JHY185 and JHY197, respectively), were inoculated at an OD of 5 and cultivated for 144 h in KP_i buffered YPD medium (pH 6.5) with or without supplementation of 2 % threonine. Culture supernatants were analysed via HPLC for 3-alkylphenol production. Error bars represent standard deviations of biological duplicates.The y-axis was truncated to visualize also small values.

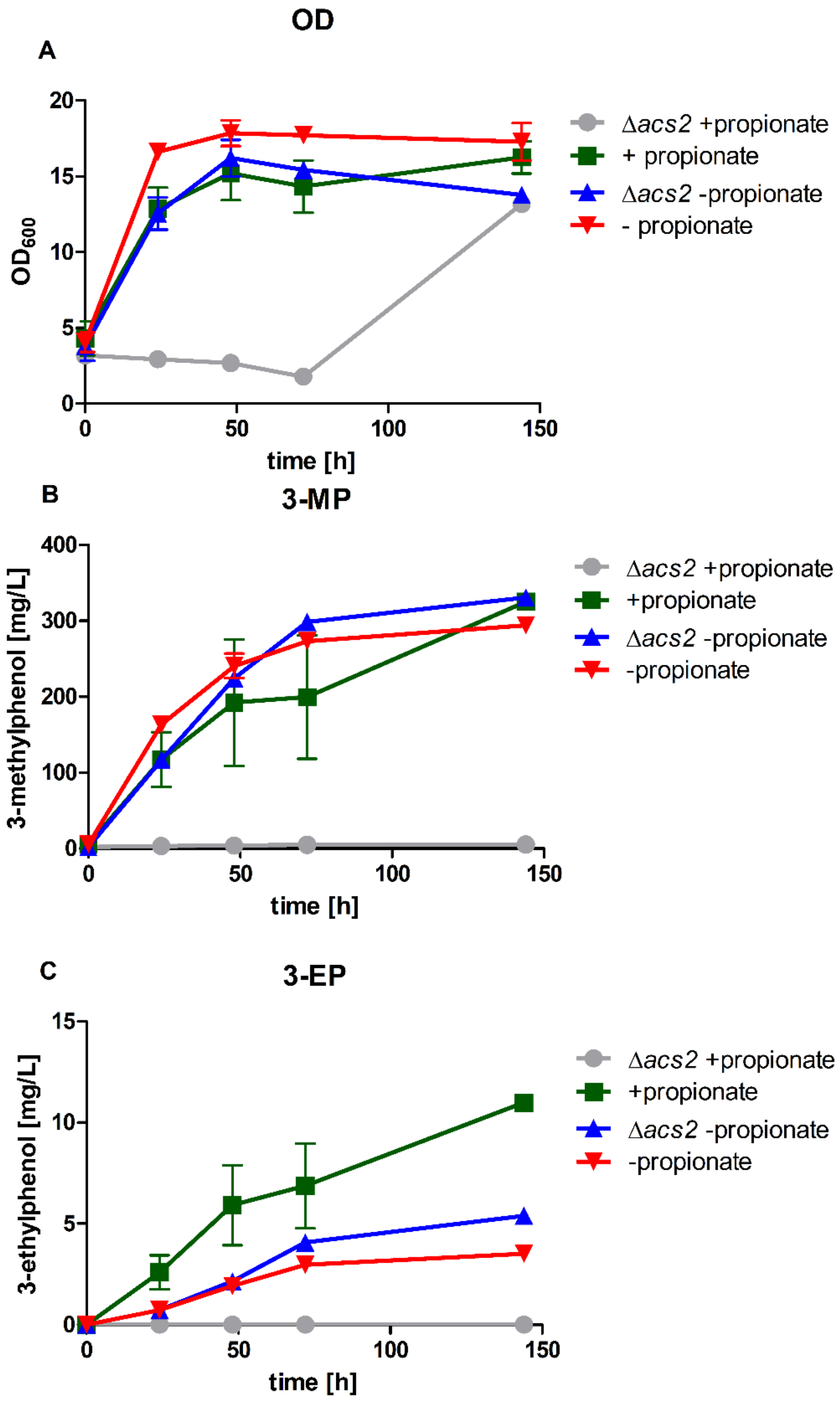

Figure S2. Influence of acs2 deletion on growth (A), 3-methylphenol (B) and 3-ethylphenol (C) formation with or without supplementation of external propionate. Yeast strains CEN.PK2-1C expressing the 3-methylphenol pathway (^{Ppopt}*MSAS*, ^{opt}*npgA* and ^{opt}*patG;* Hitschler and Boles, 2019*)*, the propionyl-CoA synthase ^{opt}*prpE*, *Δcit2Δcit3* double deletion and with or without *Δacs2* were inoculated at an OD of 3.5 and cultivated for 144 h in KP_i buffered YPD medium (pH 6.5) optionally supplemented with 10 mM propionate. Culture supernatants were analysed via HPLC for 3-alkylphenol production. Error bars represent standard deviations of biological duplicates.

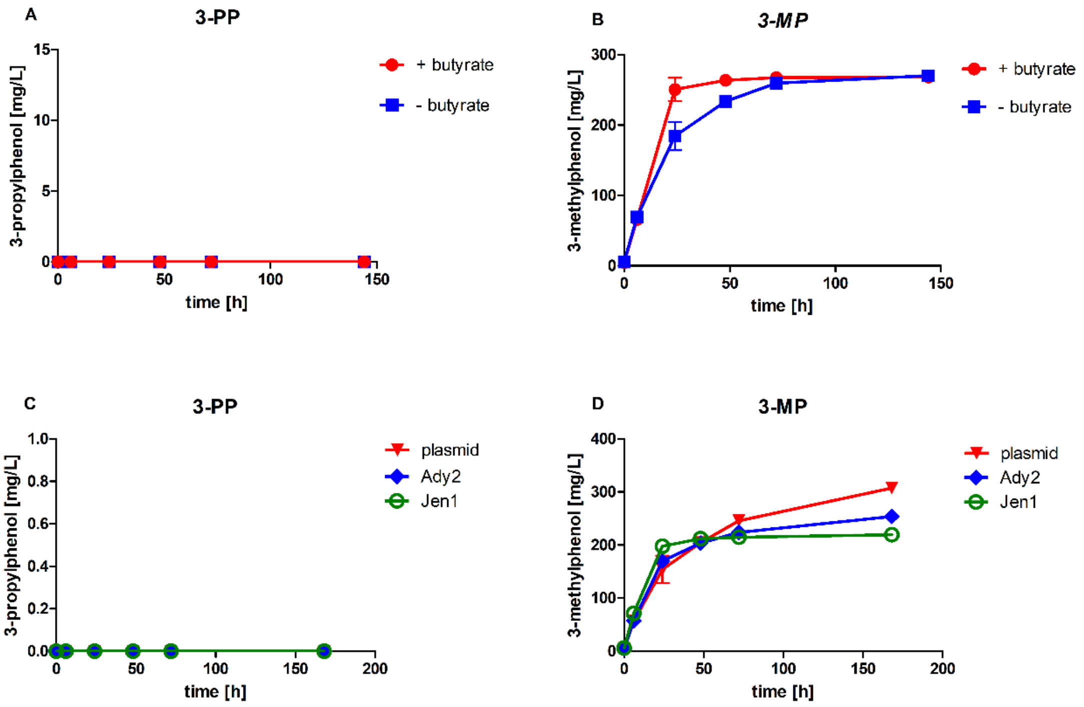

Figure S3. 3-alkylphenol production in different yeast strains with or without butyrate supplementation. 3-propylphenol (A) *and* 3-methylphenol (B) production of CEN.PK2-1C expressing the 3-methylphenol pathway (^{Ppopt}*MSAS*, ^{opt}*npgA* and ^{opt}*patG;* strain JHY162*;* Hitschler and Boles, 2019*)* with or without butyrate supplementation*.* 3-propylphenol (C) *and* 3-methylphenol (D) production of strain JHY211 (*Δpox1*) and expressing monocarboxylic acid transporters *JEN1* or *ADY2* from multi-copy plasmids pRS72N_JEN1 or pRS72N_ADY2 (empty plasmid pRS72N as control) in the presence of 10 mM butyrate. High-OD fermentations (starting OD = 4-5) were performed in biological duplicates at 30°C in KP_i buffered YPD medium (pH 6.5) supplemented with (10 mM) or without butyrate. Culture supernatants were analysed via HPLC for 3-alkylphenol concentrations. Error bars represent standard deviations.

Table S1. Primers for plasmid or strain construction used in the present work

**Primer name**	**5´-3´ sequence**	**Application**
**Deletion of *CIT2***		
ABP047_ProCIT2_fw	CATTTATCCGGTGGTCATCG	amplification of *CIT2*, forward
ABP048_terCIT2_rev	GCTAGCCAAGGCAGTAAGG	amplification of *CIT2*, reverse
ABP049_CIT2_rev	GCTTTCCAAGGCAGTTACAG	sequencing of *CIT2*, reverse
ABP054_CIT2_Del40_ Oligo	CTCAAAACTTTTTGTTTTAATAA TACTAGTAACAAGAAAATTGGAT	Donor-DNA for deletion of *CIT2* binding in *CIT2p* with overhang to *CIT2t*, forward

	TACATCCTACTTTTACACCCCTC TGCATATTTTT	
ABP055_CIT2_Del40_ Oligo_Comp	AAAAATATGCAGAGGGGTGTAA AAGTAGGATGTAATCCAATTTTC TTGTTACTAGTATTATTAAAACA AAAAGTTTTGAG	Donor-DNA for deletion of *CIT2* binding in *CIT2t* with overhang to *CIT2p*, reverse
**Cloning of pJHV54**		
MGP126_CrCASseq.fw	GGGAAACGCCTGGTATC	sequencing of gRNA, forward
WGP243_S-Cas9-1_Rv	TCTTCTTGAAGTAGTCTTCC	amplification of pRCC, reverse
WGP245_S-Cas9-3_Fw	GGCTATTGTTGACTTGTTG	amplification of pRCC, forward
JHP233_gRNA_CIT3_f	cttctgcatatatgtgcgccgTTTTAGAGC TAGAAATAGCAAGTTAAAATAAG G	amplification of pRCC with gRNA for *CIT3*, forward
JHP234_gRNA_CIT3_r	cggcgcacatatatgcagaagGATCATT TATCTTTCACTGCGGAGA	amplification of pRCC with gRNA for *CIT3*, reverse
**Deletion of *CIT3***		
JHP237_CIT3p_f	CCATGGTAGCGGTTCTAAAG	amplification of *CIT3*, forward
JHP238_CIT3t_r	TTTGTAAACGGCCCGAGG	amplification of *CIT3*, reverse
JHP239_CIT3seq_f	GGGATTAGCGGGTCCTTTG	seqencing of *CIT3*, forward
JHP240_CIT3_del40_O ligo	AGAATTTATACATAGACGCCGC TAAATAATTGAATACAAACGCAG TTCCAATTTACAAGAATGCTTCG TTTGCTATTACAA	Donor-DNA for deletion of *CIT3* binding in *CIT3p* with overhang to *CIT3t*, forward
JHP241_CIT3_del40_O ligo_comp	TTGTAATAGCAAACGAAGCATT CTTGTAAATTGGAACTGCGTTT GTATTCAATTATTTAGCGGCGT CTATGTATAAATTCT	Donor-DNA for deletion of *CIT3* binding in *CIT3t* with overhang to *CIT3p*, reverse
**Cloning of pJHV19**		
JHP108_CC_sfa1#2_r	cacagtcccgcagccaaatacGATCATT TATCTTTCACTGCGGAG	amplification of pRCC with gRNA for *SFA1*, reverse

JHP107_CC_sfa1#2_f	gtatttggctgcgggactgtgTTTTAGAGCTAGAAATAGCAAGTTAAAATAAGG	amplification of pRCC with gRNA for *SFA1*, reverse
**Genomic integration of *TDH3p-*^{Stopt}*prpE-sfa1t* into *sfa1* locus**		
JHP018_ovTDH3p_r	*ttgatagatctttggtagaattcagagaaagacatTTTGTTTGTTTATGTGTGTTTATTC*	amplification of *TDH3p* with overhang to *prpE* and pJHV1, reverse
JHP019_ovPGK1t_f	aacaaatcagacaagctatcgaagaataaccgcggATTGAATTGAATTGAAATCGATAG	amplification of *PGK1t* with overhang to *prpE* and pJHV1, forward
JHP058_prpE_ovTDH3p	tttaaaacacCAAGAACTTAGTTTCGAATAAACACACATA	amplification of *prpE* with overhang to *TDH3p* and *PGK1t,* forward
JHP059_prpE_ovPGK1t	gagaaaagaaAAAAATTGATCTATCGATTTCAATTCAATTC	amplification of *prpE* with overhang to *TDH3p* and *PGK1t,* reverse
JHP060_ovTDH3p_SacI_f	tttaatttgcggccggtacccaattcgcccgagctcACAGTTTATTCCTGGCATCCACTA	amplification of *TDH3p* with overhang to *prpE*, forward
JHP061_ovPGK1t_BamHI_r	agcgcgcaattaaccctcactaaagggaacaaggatccAAATAATATCCTTCTCGAAAGC	amplification of *PGK1t* with overhang to *prpE*, reverse
JHP075_prpE_d->c_1r	cgaagatagtgtccatagaagtAGCCAAAGCAACAGCGTAAC	correct deletion at 783 bp in *prpE*, reverse
JHP076_prpE_d->c_2f	agacgttggtggttacgctgTTGCTTTGGCTACTTCTATG	correct deletion at 783 bp in *prpE*, forward
JHP079_TDH3p_prpE_ovsfa1up_f	ataatacttacggggctagttttattttctaccacaaataaaaataaagcatcttttaaACAGTTTATTCCTGGCATCCACTA	amplification of *prpE* and *TDH3p* with overhang to *SFA1* upstream region, forward
JHP080_prpE_ovsfa1t_r	ttgattttcaaagtattccagaaaatttgagtcatgcttacttagtttaattaagtactcTTATTCTTCGATAGCTTGTCTGATT	amplification of *prpE* and *TDH3p* with overhang to *SFA1t*, reverse
vsp328_pSFA1_fw	GAATAAGTCCTGGTTCCAGGCAAAACC	primer binding upstream of *SFA1* locus to check integration, forward

MGP146_SFA1down-rev	GTTAGGAACAGGCGAGGTC	primer binding downstream of *SFA1* locus to check integration, reverse
JHP021_prpEseq1	CGCTGTTGACAGATGGAGAG	sequencing of *prpE*
JHP022_prpEseq2	CCCAGACTGTGGTGTTTG	sequencing of *prpE*
JHP083_sfa1_seq1_f	GTGACAACCGAAAGTCAG	sequencing primer binding upstream of *SFA1* locus, forward
JHP084_sfa1_seq2_r	TTTCTTCAGGTCTAACTGATTG	sequencing primer binding downstream of *SFA1* locus, reverse
**Deletion of *ACS2***		
JHP288_p-HYG-t_ovACS2_f	catatgcgtttcccggggccgaagcgttattgccgatattTTCGTACGCTGCAGGTCGAC	amplification of TEFp-hphNT1-CYC1t with overhang to upstream region of *ACS2*, forward
JHP289_p-HYG-t_ovACS2_r	cttttaccctatcccgggcgaagaaccccgtcacagtgGCATAGGCCACTAGTGGATCTG	amplification of *TEFp- hphNT1-CYC1t* with overhang to downstream region of *ACS2*, reverse
Vsp333_tACS2_rev	AACAAGGCAAAATAGCGTTAACAACC	primer binding downstream of *ACS2* locus to check deletion, reverse
Vsp334_pACS2_fw	TTTCCTGTGAGAAGTTTAAATCCACTAAGG	primer binding upstream of *ACS2* locus to check deletion, forward
Vsp338_ACS2_fw	TTGGCTGTGGCTCGTATTGGTGC	primer binding in *ACS2* locus to check deletion, forward
MGP122_hphNT1_fw	TCACTGGCAAACTGTGATGG	primer binding in *hphNT1* to check integration, forward
**Deletion of *POX1***		
ABP017*_POX1_Del40_Oligo	TCACAGAAAAAAAGAAAATATAATAAATTAGTATTGCGATGTAGAGGTTTCCTGTTTTCCTTCGAACCCTCTGTTTTGCG	Donor-DNA for deletion of *POX1* binding in *POX1p* with overhang to *POX1t*, forward
ABP025_POX1_Del40_Oligo_comp	CGCAAAACAGAGGGTTCGAAGGAAAACAGGAAACCTCTACATCGCAATACTAATTTATTATATTTTCTTTTTTTCTGTGA	Donor-DNA for deletion of *POX1* binding in *POX1t* with overhang to *POX1p*, reverse

**Assembly via part plasmids and sequencing of pJHV62 and pJHV65**		
GDP253 Rv TER_ACP1 GG as 3	CGTCTCAGGTCGGTCTCAGGATTTAAATTCTGTCGAATCTTTCAACTTC	amplification of *Ter* with overhangs for Golden Gate part 3, reverse
GDP261 Fw tdTER GG as 3	CGTCTCGTCGGTCTCATATGATTGTTAAGCCAATGGTTAGAAACAAC	amplification of *Ter* with overhangs for Golden Gate part 3, forward
GDP255 Rv Crt_mtMDH GG as 3	CGTCTCAGGTCGGTCTCAGGATTTATCTGTTCTTGAAACCTTCAATC	amplification of *Crt* with overhangs for Golden Gate part 3, reverse
GDP263 Fw Crt GG as 3	CGTCTCGTCGGTCTCATATGGAATTGAACAACGTCATC	amplification of *Crt* with overhangs for Golden Gate part 3, forward
GDP259 Rv ERG10_mtNC GG as 3	CGTCTCAGGTCGGTCTCAGGATTTAAATCTTTTCAATGACAATAGAGGAAGC	amplification of *ERG10* with overhangs for Golden Gate part 3, reverse
GDP262 Fw ERG10 GG as 3	CGTCTCGTCGGTCTCATATGTCTCAAAACGTTTACATTG	amplification of *ERG10* with overhangs for Golden Gate part 3, forward
GDP265 Rv Hbd GG as 3 part 1	CTGGGTTGAAGAAGTGCATACCAATAACCTTATCTGGCCTCTTAGTAGCAGAAG	amplification of *Hbd* part 1 without *Bsa*I cutsite and with overhangs for Golden Gate part 3, reverse
GDP266 Fw Hbd GG as 3 part 2	TCTGCTACTAAGAGGCCAGATAAGGTTATTGGTATGCACTTCTTCAAC	amplification of *Hbd* part 2 without *Bsa*I cutsite and with overhangs for Golden Gate part 3, forward
GDP267 Rv Hbd GG as 3 part 2	CGTCTCAGGTCGGTCTCAGGATTTACTTAGAGTAATCGTAGAAACCC	amplification of *Hbd* part 2 without *Bsa*I cutsite and with overhangs for Golden Gate part 3, reverse
GDP285 Fw Hbd GG as 3 part 1	CTGATTCTGTGGATAACCGTAGTCGGTCTCATATGAAGAAGGTTTGTGTTATTG	amplification of *Hbd* part 1 without *Bsa*I cutsite and with overhangs for Golden Gate part 3, forward
hdp073	GGTTGCATCACTCCATTG	sequencing primer binding in *TDH3p,* reverse
Hdp446	TCCTTTACGCTAAAATAATAGTTTATTT	sequencing primer binding in *PGK1t,* forward

JTP302	AAGGCATTAAAAGAGGAGCG	sequencing primer binding in *PGK1t,* reverse
SZ069seq1EcPPC_for	GTTATCCCCTGATTCTGTG	primer binding in SiHV110 backone, forward
vsp84_Hbd_ovpPGK1_fw	TCTACTTTTTACAACAAATATAAAACAATGAAGAAGGTTTGTGTTATTGG	sequencing primer binding in *Hbd,* forward
vsp156_seq3_tVMA16	CATACACATGTATCTCAGATATCTC	sequencing primer binding in *VMA16t,* reverse
Vsp157_seq4_ERG10	TTTCGTTGTCGAACTTACC	sequencing primer binding in *ERG10,* reverse
Vsp160_seq7_hbd	TATTGCTATTGGTAAGGATCC	sequencing primer binding in *Hbd,* forward
Vsp162_seq9_crt	CACCGAAACCTGGGG	sequencing primer binding in *Crt,* reverse
vsp313_seq55_tdTer	CCGTCTTGAAGCCATTCGG	sequencing primer binding in *Ter,* forward
vsp314_seq56_tdTer	TTACAGACACGACTTCTTGGC	sequencing primer binding in *Ter,* forward
vsp315_seq57_pTDH3	CAACTACAGAGAACAGGGGC	sequencing primer binding in *TDH3p,* forward

Table S2. Genes used in the study with their source organism and sequence. Sequences codon-optimized (opt) for *S. cerevisiae* are indicated by prefixes in superscript.

**Gene**	**Sequence**	**Source organism**
^{Ppopt}MSAS	ATGCACTCTGCTGCTACTTCTACTTACCCATCTGGTAAGACTTC<br>TCCAGCTCCAGTTGGTACTCCAGGTACTGAATACTCTGAATAC<br>GAATTCTCTAACGACGTTGCTGTTGTTGGTATGGCTTGTAGAG<br>TTGCTGGTGGTAACCACAACCCAGAATTGTTGTGGCAATCTTT<br>GTTGTCTCAAAAGTCTGCTATGGGTGAAATCCCACCAATGAGA<br>TGGGAACCATACTACAGAAGAGACGCTAGAAACGAAAAGTTCT<br>TGAAGAACACTACTTCTAGAGGTTACTTCTTGGACAGATTGGA<br>AGACTTCGACTGTCAATTCTTCGGTATCTCTCCAAAGGAAGCT<br>GAACAAATGGACCCACAACAAAGAGTTTCTTTGGAAGTTGCTT<br>CTGAAGCTTTGGAAGACGCTGGTATCCCAGCTAAGTCTTTGTC<br>TGGTTCTGACACTGCTGTTTTCTGGGGTGTTAACTCTGACGAC<br>TACTCTAAGTTGGTTTTGGAAGACTTGCCAAACGTTGAAGCTT<br>GGATGGGTATCGGTACTGCTTACTGTGGTGTTCCAAACAGAAT<br>CTCTTACCACTTGAACTTGATGGGTCCATCTACTGCTGTTGAC<br>GCTGCTTGTGCTTCTTCTTTGGTTGCTATCCACCACGGTGTTC<br>AAGCTATCAGATTGGGTGAATCTAAGGTTGCTATCGTTGGTGG<br>TGTTAACGCTTTGTGTGGTCCAGGTTTGACTAGAGTTTTGGAC	*Penicillium patulum*

	AAGGCTGGTGCTATCTCTTCTGACGGTTCTTGTAAGTCTTTCG<br>ACGACGACGCTCACGGTTACGCTAGAGGTGAAGGTGCTGGTG<br>CTTTGGTTTTGAAGTCTTTGCACAGAGCTTTGTTGGACCACGA<br>CAACGTTTTGGCTGTTATCAAGGGTTCTGCTGTTTGTCAAGAC<br>GGTAAGACTAACGGTATCATGGCTCCAAACTCTGTTGCTCAAC<br>AATTGGCTGCTAACAACGCTTTGTCTGCTGCTAACATCGACCC<br>ACACACTGTTAGATACGTTGAAGCTCACGCTACTTCTACTCCAT<br>TGGGTGACCCAACTGAAATCTCTGCTATCGCTTCTGTTTACGG<br>TGCTGACAGACCAGCTGACGACCCATGTTACATCGGTTCTATC<br>AAGCCAAACATCGGTCACTTGGAAGCTGGTGCTGGTGTTATGG<br>GTTTCATCAAGGCTGTTTTGGCTATCCAAAAGGGTGTTTTGCC<br>ACCACAAGCTAACTTGACTAAGTTGAACTCTAGAATCGACTGG<br>AAGACTGCTGGTGTTAAGGTTGTTCAAGAAGCTACTCCATGGC<br>CAGAATCTGACCCAATCAGAAGAGCTGGTGTTTGTTCTTACGG<br>TTACGGTGGTACTGTTTCTCACGCTGTTATCGAAGAATTCTCTC<br>CAATCTTGCAACCAGACCCATTGGGTAACGGTGCTGTTTCTGG<br>TCCAGGTTTGTTGTTGTTGTCTGGTCCACAAGAAAAGAGATTG<br>GCTTTGCAAGCTAAGACTTTGAGAGACTGGATGACTGCTGAAG<br>GTAAGGACCACAACTTGTCTGACATCTTGACTACTTTGGCTACT<br>AGAAGAGACCACCACGACTACAGAGCTGCTTTGGTTGTTGACG<br>ACTACAGAGACGCTGAACAAGTTTTGCAATCTTTGGCTAACGG<br>TGTTGACCACACTTTCACTACTCAATCTAGAGTTTTGGGTTCTG<br>ACATCTCTAAGGACGTTGTTTGGGTTTTCTCTGGTCACGGTGC<br>TCAATGGCCAGACATGGGTAAGCAATTGATCCACAACCCAGTT<br>TTCTTCGCTGCTATCCAACCATTGGACGAATTGATCCAAGCTG<br>AAATCGGTTTGTCTCCAATCGAATTGTTGAGAACTGGTGACTTC<br>GAATCTTCTGACAGAGTTCAAATCTTGACTTACGTTATGCAAAT<br>CGGTTTGTCTGCTTTGTTGCAATCTAACGGTATCACTCCACAA<br>GCTGTTATCGGTCACTCTGTTGGTGAAATCGCTGCTTCCGTTG<br>TTGCTGGTGCTTTGTCTCCAGCTGAAGGTGCTTTGATCGTTAC<br>TAGAAGAGCTTTGTTGTACAGACAAGTTATGGGTAAGGGTGGT<br>ATGATCTTGGTTAACTTGCCATCTGCTGAAACTGAAGAAATCTT<br>GGGTTCTAGATCTGACTTGGTTGTTGCTATCGACTCTTCTCCAT<br>CTTCTTGTGTTGTTGCTGGTGACAAGGAATTGGTTGCTGAAAC<br>TGCTGAAGCTTTGAAGGCTAGAGGTGTTAAGACTTTCACTGTT<br>AAGTCTGACATCGCTTTCCACTCTCCAACTTTGAACGGTTTGGT<br>TGACCCATTGAGAGACGTTTTGGCTGAAACTTTGTCTCCAGTTT<br>CTCCAAACGTTAAGTTGTACTCTACTGCTTTGGCTGACCCAAG<br>AGGTCAAGACTTGAGAGACGTTGAATACTGGGCTGGTAACATG<br>GTTAACAGAGTTAGATTGACTTCTGCTGTTAAGGCTGCTGTTG<br>AAGACGGTTACAGATTGTTCTTGGAAGTTTCTACTCACCCAGTT<br>GTTTCTCACTCTATCAACGAAACTTTGATGGACGCTGGTATGG<br>AAGACTTCGCTGTTATCCCAACTTTGTTGAGAAAGAAGCCAAC<br>TGAAAAGCACATCTTGCACTCTATCGCTCAATTGCACTGTAGA<br>GGTGCTGAAGTTAACTGGGCTGCTCAAATGCCAGGTAGATGG<br>GCTACTGGTGTTCCAACTACTACTTGGATGCACAAGCCAATCT<br>GGAGAAAGATCGAAACTGCTCCATTGCACACTGGTTTGACTCA<br>CGACGTTGAAAAGCACACTTTGTTGGGTCAAAGAATCCCAGTT<br>CCAGGTACTGACACTTACGTTTACACTACTAGATTGGACAACG<br>ACACTAAGCCATTCCCAGGTTCTCACCCATTGCACGGTACTGA<br>AATCGTTCCAGCTGCTGGTTTGATCAACACTTTCTTGAAGGGT<br>ACTGGTGGTCAAATGTTGCAAAACGTTGTTTTGAGAGTTCCAG<br>TTGCTATCAACGCTCCAAGATCTGTTCAAGTTGTTGTTCAACAA<br>GACCAAGTTAAGGTTGTTTCTAGATTGATCCCATCTGAACCATC<br>TCAATTGGACGACGACGCTTCTTGGGTTACTCACACTACTGCT<br>TACTGGGACAGAAAGGTTGCTGGTTCTGAAGACAGAATCGACT	

	TCGCTGCTGTTAAGTCTAGATTGGTTACTAAGTTGGCTGACAA<br>CTTCTCTATCGACTACTTGGACAAGGTTGGTGTTTCTGCTATG<br>GGTTTCCCATGGGCTGTTACTGAACACTACAGAAACGACAAGG<br>AAATGTTGGCTAGAGTTGACGTTAACCCAGCTATCTCTGGTGA<br>CGCTCCATTGCCATGGGACTCTTCTTCTTGGGCTCCAGTTTTG<br>GACGCTGCTACTTCTGTTGGTTCTACTATCTTCCCAACTCCAG<br>CTTTGAGAATGCCAGCTCAAATCGAAAGAGTTGAAGTTTTCACT<br>TCTCAAGACCCACCAAAGATCTCTTGGTTGTACGTTCAAGAAG<br>CTTCTGACTCTGTTCCAACTTCTCACGTTTCTGTTGTTTCTGAA<br>GCTGGTGAAGTTTTGGCTAAGTTCACTGCTATGAGATTCTCTG<br>AAATCGAAGGTACTCCAGGTGTTTCTGGTTCTATGGAATCTTTG<br>GTTCACCAAATCGCTTGGCCACCAGCTACTCCAGCTGAAGAAC<br>CATTGTCTATCGAAACTGTTATCTTGGTTTCTCCAGACGCTACT<br>ACTAGAGCTTTGTACGCTGCTTCTTTGCCAACTAGAGTTAACTC<br>TTTCCAATTCTCTTCTACTCAAGAATTCTTCTCTAACGCTTCTTC<br>TTTGCCATTGGAAAAGGGTACTGTTGTTACTTACATCCCAGGT<br>GAAGTTGCTTCTTTGGCTGAAGTTCCAGCTGCTTCTGAATCTTT<br>CACTTGGAACTTGTTGGAATTGATCAAGTTCACTGTTAACGGTT<br>CTTTGCCAATCAAGGTTTTCACTTTGACTGCTAACATCGGTGAA<br>GGTCAAACTCCAACTGCTTTGGCTCAATCTCCATTGTACGGTTT<br>GGCTAGAGTTATCGCTTCTGAACACCCAGACTTGGGTACTTTG<br>ATCGACGTTGAAGAACCAGTTATCCCATTGTCTACTATGAGATA<br>CATCCAAGGTGCTGACATCATCAGAATCAACGACGGTATCGCT<br>AGAACTTCTAGATTCAGATCTTTGCCAAGAAACAAGTTGTTGCC<br>AGCTTCTGAAGGTCCAAGATTGTTGCCAAGACCAGAAGGTACT<br>TACTTGATCACTGGTGGTTTGGGTGTTTTGGGTTTGGAAGTTG<br>CTGACTTCTTGGTTGAAAAGGGTGCTAGAAGATTGTTGTTGAT<br>CTCTAGAAGAGCTTTGCCACCAAGAAGAACTTGGGACCAAGTT<br>TCTGAAGACTTGCAACCAACTATCGCTAAGATCAGATTGTTGG<br>AATCTAGAGGTGCTTCTGTTCACGTTTTGCCATTGGACATCACT<br>AAGCCAGACGCTGTTGAACAATTGACTACTGCTTTGGACAGAT<br>TGTCTTTGCCATCTGTTCAAGGTGTTGTTCACGCTGCTGGTGT<br>TTTGGACAACGAATTGGTTATGCAAACTACTAGAGACGCTTTCA<br>ACAGAGTTTTGGCTCCAAAGATCGCTGGTGCTTTGGCTTTGCA<br>CGAAGTTTTCCCACCAAAGTCTGTTGACTTCTTCGTTATGTTCT<br>CTTCTTGTGGTAACTTGGTTGGTTTCACTGGTCAAGCTTCTTAC<br>GGTTCTGGTAACGCTTTCTTGGACACTTTGGCTACTCACAGAG<br>CTAGATTGGGTGACGCTGCTGTTTCTTTCCAATGGACTTCTTG<br>GAGAGGTTTGGGTATGGGTGCTTCTACTGACTTCATCAACGCT<br>GAATTGGAATCTAAGGGTATCACTGACGTTACTAGAGACGAAG<br>CTTTCGCTGCTTGGCAACACTTGGCTAAGTACGACATGGACCA<br>CGGTGTTGTTTTGAGATCTAGAGCTTTCGAAGACGGTGAACCA<br>ATCCCAGTTTCTATCTTGAACGACATCGCTGTTAGAAGAGTTG<br>GTACTGTTTCTAACACTTCTCCAGCTGCTGCTGGTTCTTCTGAC<br>GCTGTTCCAACTTCTGGTCCAGAATTGAAGGCTTACTTGGACG<br>AAAAGATCAGAGGTTGTGTTGCTAAGGTTTTGCAAATGACTGC<br>TGAAGACGTTGACTCTAAGGCTGCTTTGGCTGACTTGGGTGTT<br>GACTCTGTTATGACTGTTACTTTGAGAAGACAATTGCAACTTAC<br>TTTGAAGATCGCTGTTCCACCAACTTTGACTTGGTCTCACCCAA<br>CTGTTTCTCACTTGGCTGTTTGGTTCGCTGAAAAGTTGGCTAA<br>GTAA	

^{opt}*npgA*	ATGGTTCAAGACACTTCTTCTGCTTCTACTTCTCCAATCTTGAC<br>TAGATGGTACATCGACACTAGACCATTGACTGCTTCTACTGCT<br>GCTTTGCCATTGTTGGAAACTTTGCAACCAGCTGACCAAATCT<br>CTGTTCAAAAGTACTACCACTTGAAGGACAAGCACATGTCTTT<br>GGCTTCTAACTTGTTGAAGTACTTGTTCGTTCACAGAAACTGTA<br>GAATCCCATGGTCTTCTATCGTTATCTCTAGAACTCCAGACCCA<br>CACAGAAGACCATGTTACATCCCACCATCTGGTTCTCAAGAAG<br>ACTCTTTCAAGGACGGTTACACTGGTATCAACGTTGAATTTAAC<br>GTTTCTCACCAAGCTTCTATGGTTGCTATCGCTGGTACTGCTTT<br>CACTCCAAACTCTGGTGGTGACTCTAAGTTGAAGCCAGAAGTT<br>GGTATCGACATCACTTGTGTTAACGAAAGACAAGGTAGAAACG<br>GTGAAGAAAGATCTTTGGAATCTTTGAGACAATACATCGACATC<br>TTCTCTGAAGTTTTCTCTACTGCTGAAATGGCTAACATCAGAAG<br>ATTGGACGGTGTTTCTTCTTCTTCTTTGTCTGCTGACAGATTGG<br>TTGACTACGGTTACAGATTGTTCTACACTTACTGGGCTTTGAAG<br>GAAGCTTACATCAAGATGACTGGTGAAGCTTTGTTGGCTCCAT<br>GGTTGAGAGAATTGGAATTCTCTAACGTTGTTGCTCCAGCTGC<br>TGTTGCTGAATCTGGTGACTCTGCTGGTGACTTCGGTGAACCA<br>TACACTGGTGTTAGAACTACTTTGTACAAGAACTTGGTTGAAGA<br>CGTTAGAATCGAAGTTGCTGCTTTGGGTGGTGACTACTTGTTC<br>GCTACTGCTGCTAGAGGTGGTGGTATCGGTGCTTCTTCTAGAC<br>CAGGTGGTGGTCCAGACGGTTCTGGTATCAGATCTCAAGACC<br>CATGGAGACCATTCAAGAAGTTGGACATCGAAAGAGACATCCA<br>ACCATGTGCTACTGGTGTTTGTAACTGTTTGTCTTAA	*Aspergillus nidulans*
^{opt}*patG*	ATGGCTAAGATCGACGTTCACCACCACTTCTACCCACAAGCTA<br>TGAGAGAAGCTTTGGAAAGAGCTGGTGGTGACCCATCTGGTT<br>GGTACATCCCACCATGGACTTTGGACTTGGACAAGGAAATCTC<br>TAGAGTTTTGAAGGTTCAAACTACTATCTTGTCTGTTACTGCTC<br>CAGGTCCAGGTATCGAAACTGACCCAGGTAAGGCTGCTGCTTT<br>GGCTAGATTGTGTAACGAAGAAGCTGCTGCTATCAGAGACGCT<br>CACCCATTGCAATACGGTTTCTTCGCTTCTGTTCCATCTTTGTT<br>CGACACTGCTGCTGTTTTGGCTGAAATCGAACACGCTTTCACT<br>AACTTGCACGCTGACGGTGTTACTTTGTACACTAGATACGGTG<br>CTGGTCACTCTTACTTGGGTGACGAAAGATTCAGACCAGTTTG<br>GGCTGAATTGTCTAAGAGAAGAGCTGTTGTTTTCATCCACCCA<br>ACTCACGCTGTTGACACTCAATTGATCAACTCTTGGATGCCAC<br>AACCAATGTTCGACTACCCACACGAAACTGGTAGAACTGCTAT<br>GGACTTGTTGACTAGAGGTGTTATCAGAGACTACCCAGGTTGT<br>AAGATCATCTTGTCTCACGCTGGTGGTACTTTGCCATACTTGAT<br>CCACAGAGCTGCTACTATGTTGCCATTCATGCCAAGAAACTTG<br>GGTATGTCTAGAGAAGAAATCGTTGAAGCTGCTAGAACTTTGT<br>ACTTCGACACTGCTATCTCTGCTAACCCAGTTACTTTGAAGGCT<br>TTGTTGGAATTCGCTAAGCCAGGTCACGTTTTGTTCGGTTCTG<br>ACTTCCCAAACGCTCCAAGAGGTGCTATCACTCACTTCACTTC<br>TTTCTTGGAAGGTTACGACAACATGTCTGAAGAAACTAGAAGA<br>TTGGTTGAAAGAGAAGCTGCTTTGGAATTGTTCCCAAGATTGA<br>GAGGTCAATCTACTAGAGCTTGTTTGTAA	*Aspergillus clavatus*
^{opt}*prpE*	ATGTCTTTCTCTGAATTCTACCAAAGATCTATCAACGAACCAGA<br>AGCTTTCTGGGCTGAACAAGCTAGAAGAATCGACTGGAGACAA<br>CCATTCACTCAAACTTTGGACCACTCTAGACCACCATTCGCTA<br>GATGGTTCTGTGGTGGTACTACTAACTTGTGTCACAACGCTGT	*Salmonella typhimurium*

	TGACAGATGGAGAGACAAGCAACCAGAAGCTTTGGCTTTGATC<br>GCTGTTTCTTCTGAAACTGACGAAGAAAGAACTTTCACTTTCTC<br>TCAATTGCACGACGAAGTTAACATCGTTGCTGCTATGTTGTTGT<br>CTTTGGGTGTTCAAAGAGGTGACAGAGTTTTGGTTTACATGCC<br>AATGATCGCTGAAGCTCAAATCACTTTGTTGGCTTGTGCTAGA<br>ATCGGTGCTATCCACTCTGTTGTTTTCGGTGGTTTCGCTTCTCA<br>CTCTGTTGCTGCTAGAATCGACGACGCTAGACCAGCTTTGATC<br>GTTTCTGCTGACGCTGGTGCTAGAGGTGGTAAGATCTTGCCAT<br>ACAAGAAGTTGTTGGACGACGCTATCGCTCAAGCTCAACACCA<br>ACCAAAGCACGTTTTGTTGGTTGACAGAGGTTTGGCTAAGATG<br>GCTTGGGTTGACGGTAGAGACTTGGACTTCGCTACTTTGAGAC<br>AACAACACTTGGGTGCTTCTGTTCCAGTTGCTTGGTTGGAATC<br>TAACGAAACTTCTTGTATCTTGTACACTTCTGGTACTACTGGTA<br>AGCCAAAGGGTGTTCAAAGAGACGTTGGTGGTTACGCTGTTG<br>CTTTGGCTACTTCTATGGACACTATCTTCGGTGGTAAGGCTGG<br>TGGTGTTTTCTTCTGTGCTTCTGACATCGGTTGGGTTGTTGGT<br>CACTCTTACATCGTTTACGCTCCATTGTTGGCTGGTATGGCTA<br>CTATCGTTTACGAAGGTTTGCCAACTTACCCAGACTGTGGTGT<br>TTGGTGGAAGATCGTTGAAAAGTACCAAGTTAACAGAATGTTC<br>TCTGCTCCAACTGCTATCAGAGTTTTGAAGAAGTTCCCAACTG<br>CTCAAATCAGAAACCACGACTTGTCTTCTTTGGAAGCTTTGTAC<br>TTGGCTGGTGAACCATTGGACGAACCAACTGCTTCTTGGGTTA<br>CTGAAACTTTGGGTGTTCCAGTTATCGACAACTACTGGCAAAC<br>TGAATCTGGTTGGCCAATCATGGCTTTGGCTAGAGCTTTGGAC<br>GACAGACCATCTAGATTGGGTTCTCCAGGTGTTCCAATGTACG<br>GTTACAACGTTCAATTGTTGAACGAAGTTACTGGTGAACCATGT<br>GGTATCAACGAAAAGGGTATGTTGGTTATCGAAGGTCCATTGC<br>CACCAGGTTGTATCCAAACTATCTGGGGTGACGACGCTAGATT<br>CGTTAAGACTTACTGGTCTTTGTTCAACAGACAAGTTTACGCTA<br>CTTTCGACTGGGGTATCAGAGACGCTGAAGGTTACTACTTCAT<br>CTTGGGTAGAACTGACGACGTTATCAACATCGCTGGTCACAGA<br>TTGGGTACTAGAGAAATCGAAGAATCTATCTCTTCTTACCCAAA<br>CGTTGCTGAAGTTGCTGTTGTTGGTATCAAGGACGCTTTGAAG<br>GGTCAAGTTGCTGTTGCTTTCGTTATCCCAAAGCAATCTGACA<br>CTTTGGCTGACAGAGAAGCTGCTAGAGACGAAGAAAACGCTAT<br>CATGGCTTTGGTTGACAACCAAATCGGTCACTTCGGTAGACCA<br>GCTCACGTTTGGTTCGTTTCTCAATTGCCAAAGACTAGATCTG<br>GTAAGATGTTGAGAAGAACTATCCAAGCTATCTGTGAAGGTAG<br>AGACCCAGGTGACTTGACTACTATCGACGACCCAGCTTCTTTG<br>CAACAAATCAGACAAGCTATCGAAGAATAA	
ERG10	ATGTCTCAAAACGTTTACATTGTTTCTACTGCTAGAACCCCAAT<br>TGGTTCTTTCCAAGGTTCTTTGTCCTCCAAGACCGCTGTTGAAT<br>TGGGTGCTGTTGCTTTGAAGGGTGCTTTGGCTAAGGTTCCAGA<br>ATTGGATGCTTCCAAGGATTTCGACGAAATTATTTTCGGTAACG<br>TTTTGTCTGCTAACTTGGGTCAAGCTCCAGCTAGACAAGTTGC<br>TTTGGCTGCTGGTTTGTCTAACCACATCGTTGCTTCTACCGTTA<br>ACAAGGTCTGTGCTTCCGCTATGAAGGCTATCATTTTGGGTGC<br>TCAATCCATCAAGTGTGGTAACGCTGATGTTGTCGTTGCTGGT<br>GGTTGTGAATCTATGACTAACGCTCCATACTACATGCCAGCTG<br>CTAGAGCTGGTGCTAAGTTCGGTCAAACTGTTTTGGTTGATGG<br>TGTCGAAAGAGATGGTTTGAACGATGCTTACGATGGTTTGGCT<br>ATGGGTGTTCACGCTGAAAAGTGTGCTAGAGATTGGGATATTA<br>CTAGAGAACAACAAGACAACTTCGCTATCGAATCCTACCAAAA	*Saccharomyces cerevisiae*

	GTCTCAAAAGTCTCAAAAGGAAGGTAAGTTCGACAACGAAATT<br>GTTCCAGTTACCATTAAGGGTTTCAGAGGTAAGCCAGATACTC<br>AAGTCACCAAGGACGAAGAACCAGCTAGATTGCACGTTGAAAA<br>GTTGAGATCTGCTAGAACTGTTTTCCAAAAGGAAAACGGTACT<br>GTTACTGCTGCTAACGCTTCTCCAATCAACGATGGTGCTGCTG<br>CTGTCATCTTGGTTTCCGAAAAGGTTTTGAAGGAAAAGAACTT<br>GAAGCCATTGGCTATTATCAAGGGTTGGGGTGAAGCTGCTCAC<br>CAACCAGCTGATTTCACCTGGGCTCCATCTTTGGCTGTTCCAA<br>AGGCTTTGAAGCACGCTGGTATCGAAGACATCAACTCTGTTGA<br>TTACTTCGAATTCAACGAAGCTTTCTCTGTTGTCGGTTTGGTTA<br>ACACTAAGATTTTGAAGTTGGACCCATCTAAGGTTAACGTTTAC<br>GGTGGTGCTGTTGCTTTGGGTCACCCATTGGGTTGTTCTGGTG<br>CTAGAGTTGTTGTTACCTTGTTGTCCATCTTGCAACAAGAAGGT<br>GGTAAGATCGGTGTTGCTGCTATTTGTAACGGTGGTGGTGGTG<br>CTTCCTCTATTGTCATTGAAAAGATTTAA	
^{opt}*Hbd*	ATGAAGAAGGTTTGTGTTATTGGTGCTGGTACTATGGGTTCTG<br>GTATTGCTCAAGCTTTCGCTGCTAAGGGTTTCGAAGTTGTTTTG<br>AGAGATATTAAGGATGAATTCGTTGATAGAGGTTTGGATTTCAT<br>CAACAAGAACTTGTCTAAGTTGGTTAAGAAGGGTAAGATTGAA<br>GAAGCTACTAAGGTTGAAATCTTGACTAGAATTTCCGGTACCG<br>TTGACTTGAACATGGCTGCTGATTGTGATTTGGTTATTGAAGCT<br>GCTGTTGAAAGAATGGATATTAAGAAGCAAATTTTCGCTGACTT<br>GGACAACATTTGTAAGCCAGAAACCATTTTGGCTTCTAACACCT<br>CTTCTTTGTCTATTACCGAAGTTGCTTCTGCTACTAAGAGGCCA<br>GATAAGGTTATTGGTATGCACTTCTTCAACCCAGCTCCAGTTAT<br>GAAGTTGGTTGAAGTTATTAGAGGTATTGCTACCTCTCAAGAAA<br>CTTTCGATGCTGTTAAGGAAACCTCTATTGCTATTGGTAAGGAT<br>CCAGTTGAAGTTGCTGAAGCTCCAGGTTTCGTTGTTAACAGAA<br>TTTTGATTCCAATGATTAACGAAGCTGTTGGTATTTTGGCTGAA<br>GGTATTGCTTCTGTTGAAGACATTGATAAGGCTATGAAGTTGG<br>GTGCTAACCACCCAATGGGTCCATTGGAATTGGGTGATTTCAT<br>TGGTTTGGATATTTGTTTGGCTATTATGGATGTTTTGTACTCTG<br>AAACTGGTGATTCTAAGTACAGACCACACACCTTGTTGAAGAA<br>GTACGTTAGAGCTGGTTGGTTGGGTAGAAAGTCTGGTAAGGG<br>TTTCTACGATTACTCTAAGTAA	*Clostridium acetobutylicum*
^{opt}*Crt*	ATGGAATTGAACAACGTCATCTTGGAAAAGGAAGGTAAGGTTG<br>CTGTTGTTACCATTAACAGACCAAAGGCTTTGAACGCTTTGAAC<br>TCTGATACCTTGAAGGAAATGGATTACGTTATTGGTGAAATTGA<br>AAACGATTCTGAAGTTTTGGCTGTTATTTTGACTGGTGCTGGTG<br>AAAAGTCTTTCGTTGCTGGTGCTGATATTTCTGAAATGAAGGAA<br>ATGAACACCATTGAAGGTAGAAAGTTCGGTATTTTGGGTAACA<br>AGGTTTTCAGAAGATTGGAATTGTTGGAAAAGCCAGTTATTGCT<br>GCTGTTAACGGTTTCGCTTTGGGTGGTGGTTGTGAAATTGCTA<br>TGTCTTGTGATATTAGAATTGCTTCTTCTAACGCTAGATTCGGT<br>CAACCAGAAGTTGGTTTGGGTATTACCCCAGGTTTCGGTGGTA<br>CCCAAAGATTGTCTAGATTGGTTGGTATGGGTATGGCTAAGCA<br>ATTGATTTTCACTGCTCAAAACATTAAGGCTGATGAAGCTTTGA<br>GAATCGGTTTGGTTAACAAGGTTGTTGAACCATCTGAATTGAT<br>GAACACCGCTAAGGAAATTGCTAACAAGATTGTTTCTAACGCT<br>CCAGTTGCTGTTAAGTTGTCTAAGCAAGCTATTAACAGAGGTAT	*Clostridium acetobutylicum*

	GCAATGTGATATTGATACTGCTTTGGCTTTCGAATCTGAAGCTT<br>TCGGTGAATGTTTCTCTACCGAAGATCAAAAGGATGCTATGAC<br>CGCTTTCATTGAAAAGAGAAAGATTGAAGGTTTCAAGAACAGA<br>TAA	
^{opt}*Ter*	ATGATTGTTAAGCCAATGGTTAGAAACAACATTTGTTTGAACGC<br>TCACCCACAAGGTTGTAAGAAGGGTGTTGAAGATCAAATTGAA<br>TACACCAAGAAGAGAATTACCGCTGAAGTTAAGGCTGGTGCTA<br>AGGCTCCAAAGAACGTTTTGGTTTTGGGTTGTTCTAACGGTTA<br>CGGTTTGGCTTCTAGAATTACCGCTGCTTTCGGTTACGGTGCT<br>GCTACTATCGGTGTTTCCTTCGAAAAGGCTGGTTCTGAAACCA<br>AGTACGGTACCCCAGGTTGGTACAACAACTTGGCTTTCGACGA<br>AGCTGCTAAGAGAGAAGGTTTGTACTCCGTTACTATTGACGGT<br>GACGCTTTCTCCGATGAAATCAAGGCTCAAGTTATCGAAGAAG<br>CTAAGAAGAAGGGTATTAAGTTCGATTTGATTGTTTACTCTTTG<br>GCTTCTCCAGTTAGAACCGATCCAGACACCGGTATTATGCACA<br>AGTCCGTCTTGAAGCCATTCGGTAAGACCTTCACCGGTAAGAC<br>CGTCGATCCATTCACCGGTGAATTGAAGGAAATCTCCGCTGAA<br>CCAGCTAACGATGAAGAAGCTGCTGCTACCGTTAAGGTTATGG<br>GTGGTGAAGACTGGGAAAGATGGATTAAGCAATTGTCTAAGGA<br>AGGTTTGTTGGAAGAAGGTTGTATTACCTTGGCTTACTCCTACA<br>TCGGTCCAGAAGCTACTCAAGCTTTGTACAGAAAGGGTACCAT<br>TGGTAAGGCTAAGGAACACTTGGAAGCTACTGCTCACAGATTG<br>AACAAGGAAAACCCATCTATTAGAGCTTTCGTTTCTGTTAACAA<br>GGGTTTGGTTACCAGAGCTTCTGCTGTTATCCCAGTTATTCCAT<br>TGTACTTGGCTTCCTTGTTCAAGGTTATGAAGGAAAAGGGTAA<br>CCACGAAGGTTGTATCGAACAAATTACCAGATTGTACGCTGAA<br>AGATTGTACAGAAAGGACGGTACCATCCCAGTCGATGAAGAAA<br>ACAGAATCAGAATCGACGACTGGGAATTGGAAGAAGACGTTCA<br>AAAGGCTGTTTCTGCTTTGATGGAAAAGGTTACCGGTGAAAAC<br>GCTGAATCTTTGACCGACTTGGCTGGTTACAGACACGACTTCT<br>TGGCTTCTAACGGTTTCGATGTTGAAGGTATCAACTACGAAGC<br>TGAAGTTGAAAGATTCGACAGAATTTAA	*Treponema denticola*
